# Quality Improvement for Cardiovascular Disease Care in Low- and Middle-Income Countries: A Systematic Review

**DOI:** 10.1371/journal.pone.0157036

**Published:** 2016-06-14

**Authors:** Edward S. Lee, Rajesh Vedanthan, Panniyammakal Jeemon, Jemima H. Kamano, Preeti Kudesia, Vikram Rajan, Michael Engelgau, Andrew E. Moran

**Affiliations:** 1 Department of Medicine, Division of Geriatric, Hospital, Palliative and General Internal Medicine, Keck School of Medicine of University of Southern California, Los Angeles, California, United States of America; 2 Department of Medicine, Division of Cardiology, Icahn School of Medicine at Mount Sinai, New York, New York, United States of America; 3 Centre for Control of Chronic Conditions, Public Health Foundation of India, Kerala, India; 4 Moi University College of Health Sciences, Eldoret, Kenya; 5 Academic Model Providing Access to Healthcare, Eldoret, Kenya; 6 Health, Nutrition and Population Global Practice, The World Bank, Kathmandu, Nepal; 7 The World Bank, Jakarta, Indonesia; 8 Center for Translation Research and Implementation Science, National Heart Lung and Blood Institute, National Institutes of Health, Bethesda, Maryland, United States of America; 9 Department of Medicine, Division of General Medicine, Columbia University Medical Center, New York, New York, United States of America; University of Perugia, ITALY

## Abstract

**Background:**

The majority of global cardiovascular disease (CVD) burden falls on people living in low- and middle-income countries (LMICs). In order to reduce preventable CVD mortality and morbidity, LMIC health systems and health care providers need to improve the delivery and quality of CVD care.

**Objectives:**

As part of the Disease Control Priorities Three (DCP3) Study efforts addressing quality improvement, we reviewed and summarized currently available evidence on interventions to improve quality of clinic-based CVD prevention and management in LMICs.

**Methods:**

We conducted a narrative review of published comparative clinical trials that evaluated efficacy or effectiveness of clinic-based CVD prevention and management quality improvement interventions in LMICs. Conditions selected *a priori* included hypertension, diabetes, hyperlipidemia, coronary artery disease, stroke, rheumatic heart disease, and congestive heart failure. MEDLINE and EMBASE electronic databases were systematically searched. Studies were categorized as occurring at the system or patient/provider level and as treating the acute or chronic phase of CVD.

**Results:**

From 847 articles identified in the electronic search, 49 met full inclusion criteria and were selected for review. Selected studies were performed in 19 different LMICs. There were 10 studies of system level quality improvement interventions, 38 studies of patient/provider interventions, and one study that fit both criteria. At the patient/provider level, regardless of the specific intervention, intensified, team-based care generally led to improved medication adherence and hypertension control. At the system level, studies provided evidence that introduction of universal health insurance coverage improved hypertension and diabetes control. Studies of system and patient/provider level acute coronary syndrome quality improvement interventions yielded inconclusive results. The duration of most studies was less than 12 months.

**Conclusions:**

The results of this review suggest that CVD care quality improvement can be successfully implemented in LMICs. Most studies focused on chronic CVD conditions; more acute CVD care quality improvement studies are needed. Longer term interventions and follow-up will be needed in order to assess the sustainability of quality improvement efforts in LMICs.

## Introduction

Cardiovascular disease (CVD) is the leading cause of death globally. While the number of CVD deaths has not changed significantly in high-income countries since 1990, the number of deaths has increased by about 66% in low- and middle-income countries (LMICs) over the same interval[1]. While attempting to address the burden of CVD, LMICs face the challenges of limited health care budgets and infrastructure as well as constrained professional health workforce capacity.

In the context of scarce resources available to support health care in LMICs, the imperative of clinical quality improvement is to *get the most out of known effective interventions within the limitations of available resources* rather than recommending unproven interventions that would require early phase studies or those that would require substantial financial and human resource investment for implementation. Clinical quality improvement can be implemented within any setting immediately, and need not be expensive.

Quality care delivery in lower resource settings does not necessarily mean dissemination and implementation of a universal set of standards formulated in high-income countries. Standards and interventions should be dictated by context and community capacity. Adaptation to local settings is necessary to achieve optimal clinical outcomes and patient satisfaction.

As part of the Disease Control Priorities Three (DCP3) Study efforts addressing quality improvement, we reviewed and narratively summarized currently available evidence on quality improvement for CVD prevention and management in LMICs. In keeping with the Institute of Medicine definition of clinical care quality, we focused on studies that compared specific clinical care quality improvement interventions with usual care and specified measurable health outcomes[2].

## Materials and Methods

**Fig 1:**
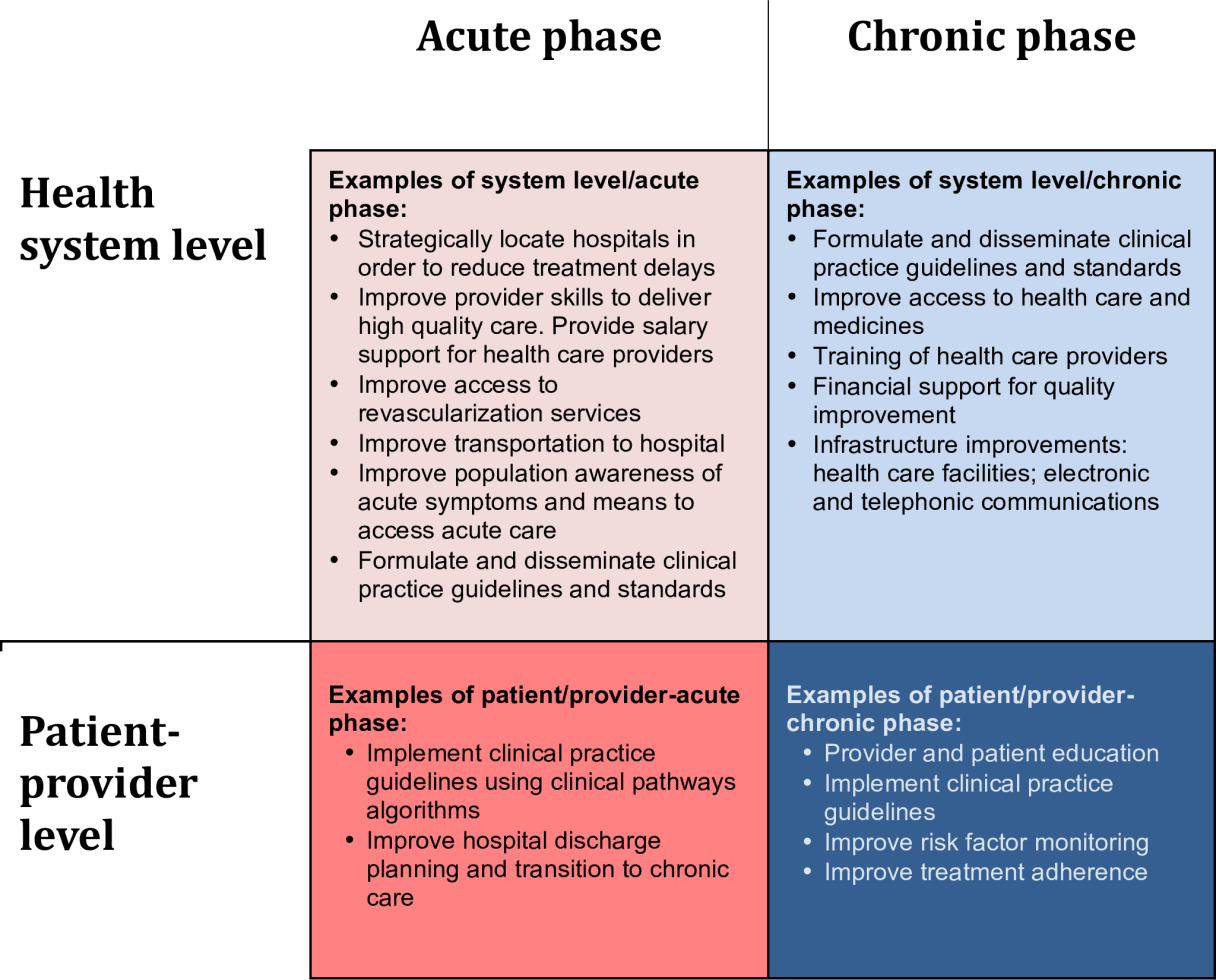
Quality conceptual framework for cardiovascular diseases, the DCP3.

A conceptual framework guided the systematic review. We specified four domains, cross-cutting between two distinct phases of CVD (acute versus chronic) and two levels of intervention (health system versus patient/provider) (Fig 1). Acute phases of CVD, such as acute myocardial infarction, stroke, and limb ischemia, occur unpredictably. Good outcomes demand timely clinical responses, which require adequate and accessible facilities, functional transportation networks, providers prepared to treat cases that present at all hours, and patient awareness of when and how to seek medical attention. In contrast, chronic phases of CVD, such as diabetes mellitus, hypertension, and congestive heart failure (CHF), require screening for pre-clinical risk factors, systematic monitoring for complications, and substantial patient self-care and engagement in order to initiate and maintain treatment adherence. Good quality chronic phase care may prevent or delay onset of acute phase manifestations, thereby preventing or delaying disability or death. We also examined interventions at the health care system and patient/provider levels. Our team populated the four domains of this two-by-two framework *a priori* with potential quality improvement levers, based on our prior knowledge of the field and examples gleaned from other studies, including other DCP3 reviews.

### Information Sources and Search Strategy

Two authors (EL and AEM) designed an electronic search of MEDLINE and EMBASE electronic databases for the purpose of capturing published reports of CVD care quality improvement studies carried out in LMICs and published in English between January 2000 and June 2014. Pertinent conditions selected for the search were myocardial infarction, acute coronary syndrome (ACS), hypertension, cardiovascular disease, diabetes, stroke, cerebrovascular disease, obesity, CHF, and rheumatic heart disease (RHD). Based on key words identified in an informal review of publications already known to the authors and a survey of the other DCP3 chapter authors, a second set of search terms related to quality improvement was identified (S1 and S2 Tables). Study designs were limited to controlled clinical trials, comparison studies, systematic reviews, and meta-analyses. Studies were limited to those published in peer-reviewed journals or in reports from institutes with rigorous internal reviews. The electronic search was refined and structured by a research librarian. Search results were considered valid if all of five key papers identified a priori by coauthors’ review were retrieved by the electronic search[3–7]. The review adhered to PRISMA recommendations for the conduct and reporting of systematic reviews (S1 Fig).

### Eligibility Criteria and Study Selection

Two authors (EL and AEM) independently assessed identified studies for eligibility based on the title and abstract. Selected full text papers were then independently reviewed by both authors. Eligibility criteria included: 1) LMIC setting; 2) patient/provider or system level intervention aimed at improving CVD quality of care; 3) outcome included risk factor or disease screening, case-finding, clinic-based CVD prevention (medication adherence, clinic visits, vital signs, laboratory results, mortality), behavioral changes (diet or exercise), improved access to medical care, or clinical guideline implementation; and 4) study design involved a comparison between a clinical quality improvement intervention and usual care, regardless of whether the intervention group served as its own control (“before and after” study) or a separate control group was used.

### Data Collection/Data Items/Summary Measures

Data were abstracted and each study was categorized into one or more quadrants of the chapter rubric (Fig 1). The following information was extracted from each study: study characteristics (setting, targeted disease, study design, participant number and baseline characteristics), type of intervention, observation interval, and outcome measures. The main summary measures were differences in means, proportions of the outcome, and outcome risk reduction. Three study types were observed most frequently and were therefore identified for potential pooling in a quantitative analysis: task-shifting in hypertension control, mobile health in diabetes management, and ACS clinical pathway interventions. Four out of eight task-shifting hypertension control studies did not report variance around the most common main effect estimate—systolic blood pressure (SBP) change [8–15]. Only three out of six studies analyzing mobile health in diabetes management reported variance around mean hemoglobin A1c (HbA1c) change [16–21]. Too few studies reported on changes in fasting plasma glucose or percent adherence to medications. ACS clinical pathways studies did not consistently report on the same quality improvement metrics [3, 5, 22]. Because of heterogeneity of outcomes and lack of reporting on measures of dispersion about main effect estimates (variance), the authors were unable to perform a quantitative analysis.

## Results

### Study Selection and Characteristics

**Fig 2:**
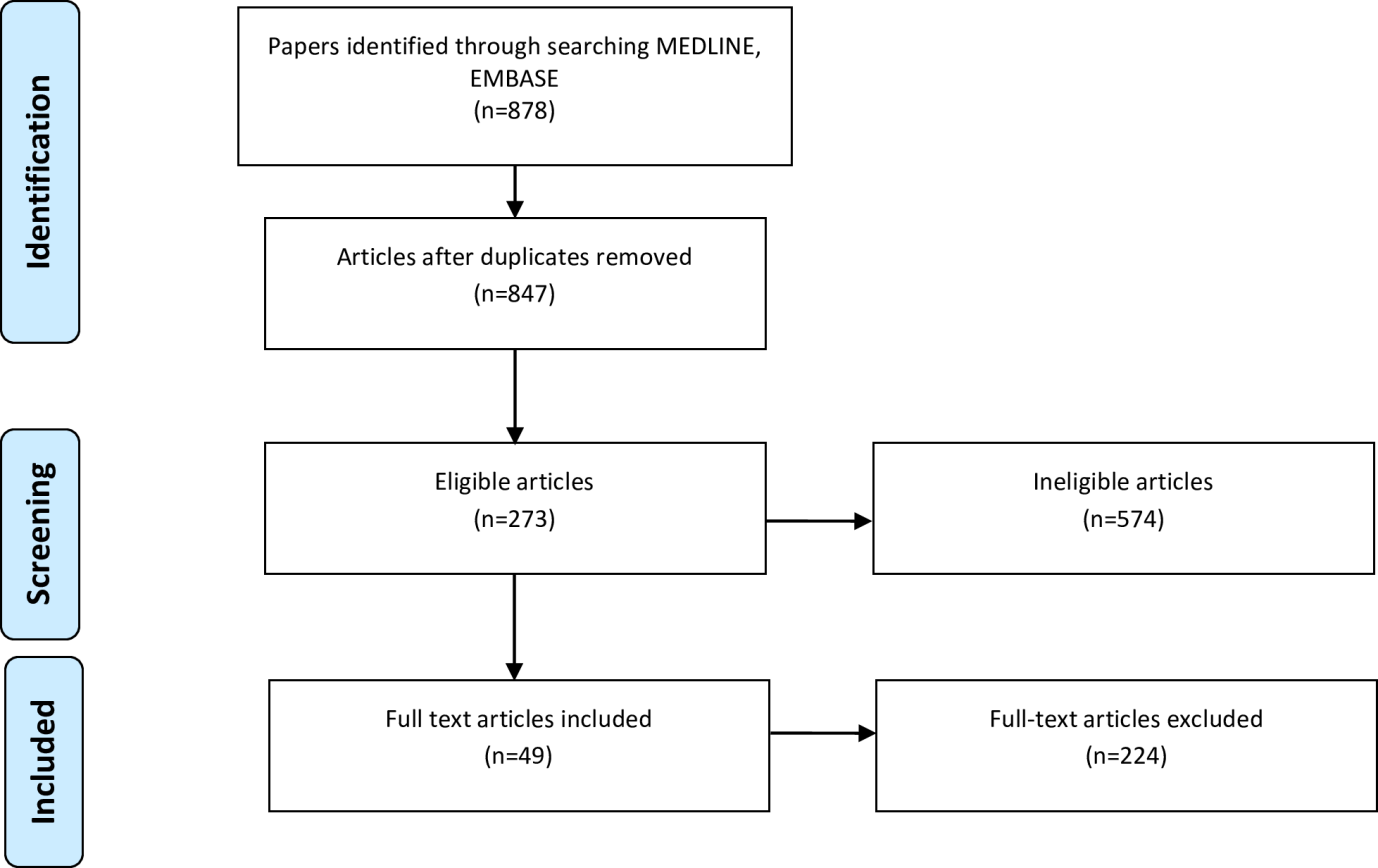
Flow diagram describing the systematic literature review process.

The search yielded 847 unique references and all five key papers identified *a priori* were captured. Based on a review of titles and abstracts, 273 potentially eligible papers were identified. Of these, two authors (EL and AEM) concurred that 49 papers reported population-based studies with clinically meaningful outcomes. The papers were selected for the detailed review (Fig 2). Table 1 presents a summary of study characteristics. All 49 studies are reported in Tables 2–5.

**Table 1:** Study Characteristics (n = 49).

	System level-acute phase	System level-chronic phase	Patient/provider level-acute phase	Patient/provider level-chronic phase	Total
**LMIC Region**					
Africa	0	4	0	7*	11*
Asia	2**	1	3**	13*	18*
Eastern Europe	0	0	1	1	2
Latin America	1	3	1	6	11
Middle East	0	0	0	8	8
**Disease Type**					
Acute coronary syndrome	3**	0	4**	1	7^a^
Congestive heart failure	0	0	0	4	4^b^
Diabetes	0	2	0	12	14^c^
Hypertension	0	3	0	17	20^d^
Rheumatic heart disease	0	2	0	0	2^e^
General CVD	0	1	0	0	1^f^
Stroke	0	0	1	0	1^g^
**TOTAL**	**3**	**8**	**5**	**34**	**49**

*Mendis et al. counted twice since study conducted in Africa (Nigeria) and Asia (China)

**Prabhakaran et al. counted for both system level and patient/provider level interventions

a-Asia (4), Latin America (2), Europe (1)

b-Latin America (3), Europe (1)

c-Asia (5), Middle East (4), Latin America (3), Asia (2)

d-Asia (7), Africa (5), Middle East (4), Latin America (3), and Africa/Asia (1)

e-Africa (2)

f-Africa (1)

g-Asia (1)

**Table 2:** System level-acute phase CVD quality improvement studies identified during review.

Quality Improvement Intervention	Study Authors	Country	Study Design	Sample	Observation Interval	Quality Measures	Results
Education program for physicians and community members on detection and optimal management of ACS	Prabhakaran et al.[22]	India	Prospective, non-randomized study; 34 hospitals treating ACS patients in Kerala region; 629 ACS patients pre-intervention and 403 post-intervention	1033 ACS patients; mean age 58; male 71–78%;	Follow-up: inpatient hospitalization	Use of aspirin, heparin, beta blockers, lipid lower agents, calcium channel blockers (CCB), time-to-thrombolysis	Absolute decreases of 43 minutes in symptom-to-door time (p<0.05) and 11 minutes in door-to-thrombolysis time (p<0.05). Total decrease in time-to-thrombolysis of 55 minutes. Significant increase in use of aspirin, heparin, beta blockers, lipid-lowering agents. Reduction in CCB use
Organize hospitals in “hub-and-spoke” model	Alexander et al.[23]	India	Prospective, multicenter, community based study	Plan to enroll 1,500 consecutive ST-elevation myocardial infarction patients at participating institutions	Patients will be enrolled over 9 months and each followed for one year	“Before and after” study of the use of reperfusion therapy, time-to-reperfusion, rates of coronary angiography within 3 to 24 hours of fibrinolytic therapy	Not yet available
National health care reform (AUGE)	Nazzal et al.[24]	Chile	Retrospective, multicenter	ST-elevation myocardial infarction patients from ten hospitals that perform thrombolysis as main perfusion therapy; 2623 pre-intervention and 906 post-intervention	N/A	Global in-hospital mortality and in patients treated with thrombolytics, evidence-based prescribing	10% absolute increase in use of thrombolysis (50% vs 60.5%). 3.4% absolute reduction in global in-hospital mortality (8.6% vs 12.0%, p<0.003) and 3.8% in patients treated with thrombolytics (6.8% vs 10.6%, p<0.005). Adjusted odds ratio (OR) for in-hospital mortality was 0.64 [95% CI 0.47,0.86]

**Table 3:** System level-chronic phase CVD quality improvement studies identified during review.

Quality Improvement Intervention	Study Authors	Country	Study Design	Sample	Observation interval	Quality Measures	Results
Enrollment in health insurance, *“Seguro Popular”*	Bleich et al.[25]	Mexico	Cross-sectional, 2005 Mexican national health and nutritional surveys	Participants with hypertension; 1065 insured matched with 1065 uninsured patients; 37% males	Survey conducted three years after start of *“Seguro Popular”*	Self-reported hypertension treatment and control	Higher probability of receiving hypertensive treatment (OR 1.5 [1.27,1.78]) and control of BP (OR 1.49 [1.00,2.20])
Enrollment in health insurance, *“Seguro Popular”*	Sosa-Rubi et al.[26]	Mexico	Cross-sectional, ENSANUT national survey in 2005–2006	Adults with diabetes; 425 insured matched with 1029 uninsured patients	N/A	Access outcomes (physician visits, treatment, weekly insulin injections, blood glucose tests) and biological outcomes (HbA1c)	Adults enrolled in *Seguro Popular* were more likely to have some type of blood glucose control test (average treatment effect on the treated (ATT) 0.095 [0.02,0.16]) and also to have appropriate glucose control (ATT 0.056 [0.009,0.103])
Community based health insurance	Hendriks et al.[27]	Nigeria	Prospective, non-randomized, non-blinded. 1 geographic area with intervention, 1 control area	Adults with hypertension; 313 in intervention area and 251 in control area; median age 55 (control) and 60 (intervention); 29% males in intervention	Intervention and follow-up: 1 year	BP measured by trained interviewers	SBP decreased by 10.4 vs 5.24mm Hg in intervention arm (p = 0.02). DBP decreased by 4.3 vs 2.2mm Hg in in intervention arm (p = 0.04)
Medication subsidy program providing full coverage of anti-hypertension medications	Yu et al.[28]	Rural China	Prospective cohort study with propensity-score matched controls	Low-income, hypertensive adults taking >1 anti-hypertensive medication (93% taking >3 meds); 102 in intervention matched with 102 controls;	Intervention and follow-up: 18 months	BP, medication adherence, and health care costs	Intervention arm had a 9% absolute increase in medication adherence (75% vs 66%, p = 0.034) and lower annual out-of-pocket medical costs both overall (combined inpatient and outpatient services, and medications) and outpatient services (p<0.001 for both)
Non-lab-based CVD score	Gaziano et al.[29]	South Africa	Cross sectional, 13 data sets (i.e. PURE, CRIBSA, etc.) from 1987–2009	14772 surveyed adults; 25–74 years old	N/A	Spearman correlation coefficient when compared to 6 lab-based scores (3 versions of Framingham, SCORE for high- and low-risk countries, and CUORE)	Non-lab-based score was closely correlated with lab-based score (Spearman 0.88–0.986); 18% of South African adults found to have high CVD risk (10-year CVD death risk >20%)
Screening echocardiogram	Beaton et al.[30]	Uganda	Prospective, non-randomized, school-based	69 healthy schoolchildren, served as own controls, mean age 5–16	Intervention: immediate. Follow-up: none	Detection of RHD	Echocardiogram detected 3 times more than auscultation (1.5% vs 0.5%, p<0.001)
Screening echocardiogram	Carapetis et al.[31]	Tonga	Prospective, non-randomized, school-based	5053 healthy schoolchildren; served as own controls, age 4–12	Intervention: immediate. Follow-up: none	Detection of RHD	Auscultation is poorly sensitive (46%) for RHD
Health insurance	Trujillo et al.[32]	Colombia	Cross-sectional, 2007 Colombian National Health Survey	Diabetic adults; 662 in contributory system, 588 in subsidized system and 188 uninsured	N/A	Preventative services utilization	No moral hazard (decrease in preventative services) in diabetic patients after introduction of health insurance

**Table 4:** Patient/provider level-acute phase CVD quality improvement studies identified during review.

Quality Improvement Intervention	Study Authors	Country	Study Design	Sample	Observation Interval	Quality Measures	Results
**Coronary artery disease**							
Multifaceted quality improvement intervention with educational materials, reminders, algorithms, and training visits	Berwanger et al.[5]	Brazil	Prospective, cluster-randomized, controlled, multicenter study; major, general public, urban hospitals with emergency departments; 17 hospitals randomized to intervention and 17 to routine practice; concealed allocation	1150 ACS patients in 34 public hospitals; mean age 62; male 69%	Follow-up: 30 days	Evidence-based therapy (aspirin, clopidogrel, anticoagulants, and statins) for ACS within first 24 hours	Significant improvement in intervention group (67.9% vs 49.5%, p = 0.01, OR 2.64 [1.28,5.45]). Intervention group also more likely to receive all eligible acute and discharge medications (50.9% vs 31.9%, p = 0.03, OR 2.49 [1.08,5.74]) and higher adherence. But no change in 30-day all-cause mortality or in-hospital cardiovascular events.
ACC/AHA approved clinical pathways	Du et al.[3]	China	Prospective, cluster-randomized, controlled, multicenter study; regional and tertiary, urban hospitals with >100 ACS patients annually; 32 hospitals to early intervention and 38 hospitals to late intervention	3500 ACS patients; mean age 64; male 67–72%	Follow-up: inpatient hospitalization	Primary outcomes were correct final diagnosis, thrombolysis or percutaneous coronary intervention within 12 hours, door-to-needle time, door-to-balloon time, high-risk patients undergoing angiography, low-risk patients undergoing functional testing, discharge on correct medications, and hospital length of stay.	12% absolute higher discharge on recommended therapies (relative risk (RR) 1.23 [1.06,1.42]). No difference in other primary outcomes, death, or major cardiovascular events.
Education program for physicians and community members on detection and optimal management of ACS	Prabhakaran et al.[22]	India	Prospective, non-randomized study; 34 hospitals treating ACS patients in Kerala region; 629 ACS patients pre-intervention and 403 post-intervention	1033 ACS patients; mean age 58; male 71–78%;	Follow-up: inpatient hospitalization	Use of aspirin, heparin, beta blockers, lipid lower agents, CCB, time-to-thrombolysis	Absolute decreases of 43 minutes in symptom-to-door time (p<0.05) and 11 minutes in door-to-thrombolysis time (p<0.05). Total decrease in time-to-thrombolysis of 55 minutes. Significant increase in use of aspirin, heparin, beta blockers, lipid lowering agents. Reduction in CCB use
Establishing a quality improvement program with a senior cardiologist	Flather et al. [33]	Poland	Prospective, cluster-randomized, multicenter, multinational study; 19 hospitals to intervention and 19 to control	874 non-ST elevation myocardial infarction or unstable angina subjects in Poland; mean age 65 (total group); male 70% (total group)	Follow-up: inpatient hospitalization	8 in-hospital quality measures (risk stratification, coronary angiography, anticoagulation, beta blockers, statins, angiotensin-converting enzyme inhibitor (ACEI), clopidogrel loading and clopidogrel at discharge	Significant improvement in primary outcome (OR 2.37 [1.76,3.2])
**Stroke**							
Guideline based structured case program for secondary stroke prevention	Peng et al.[34]	China	Prospective, cluster-randomized, multicenter study; large regional or tertiary hospitals; 23 hospitals to intervention and 24 to control	1287 inpatient stroke patients; mean age 60–61; male 67–69%	Follow-up: 12 months	Medication adherence to secondary prevention	Higher adherence to statins (56% vs 33%, p = 0.006). No difference in antiplatelet, antihypertensive, or diabetes drugs. No difference in composite end-point (new stroke, ACS, and all-cause death)

**Table 5:** Patient/provider level-chronic phase CVD quality improvement studies identified during review.

Quality Improvement Intervention	Study Authors	Country	Disease	Study Design	Sample	Observation Interval	Quality Measures	Results
**Combination pills**								
Fixed-dose combination medications	Thom et al.[8]	India	CVD/Secondary prevention	Prospective, randomized, open label, multicenter, multinational, blinded end-point trial; 501 patients to intervention and 499 to usual care	Participants with CVD or five-year CVD risk > = 15%; mean age 62 years; about 80% males	Median intervention and follow-up: 15 months	Self-reported adherence to all of aspirin, statin, and 2 or more antihypertensive medications	25.5% absolute higher adherence to all four medications (89.1% vs 63.6%, risk ratio of 1.4 [1.3–1.51]). Small but statistically significant decreases in SBP (3.8mm Hg [1.8,5.9]) and low-density lipoprotein (LDL) (10.8 mg/dL [7.4,14.1])
Low-dose combination pill, “Polycap”	Yusuf et al. [9]	India	CVD	Prospective, double blinded, multicenter trial; 2053 individuals randomized to 8 groups; 412 to intervention, ~200 to each of 8 groups	Subjects aged 45–80 years without previous CVD but with known risk factors	Intervention: 12 weeks. Follow-up: 16 weeks	BP, LDL, heart rate, urinary 11-dehydrothromboxane B2	Significant reductions in SBP and DBP by 7.4mm Hg [6.1,8.1] and 5.6mm Hg [4.7,6.4] compared to groups not receiving anti-hypertensives. Significant reduction in LDL by 0.7mmol/L [0.62,0.78] compared to groups without simvastatin
Full-dose with potassium vs low-dose combination pills (includes hydrochlorothiazide, atenolol, ramipril, simvastatin, and aspirin)	Yusuf et al.[35]	India	CVD	Prospective, randomized, multicenter trial; 257 patients to full-dose and 261 to low-dose	Subjects with >40 years of age; 2 criteria: 1) BP > 130/90 on 2 consecutive occasions or on antihypertensive medications 2) vascular disease or high-risk diabetes mellitus	Intervention: 8 weeks Follow-up: 12 weeks	BP, heart rate, serum lipids, serum and urinary potassium, and tolerability	Significant reductions in SBP and DBP by 2.8mm Hg (p = 0.003) and 1.7mm Hg (p = 0.001). Significant reductions in both total cholesterol (p = 0.014) and LDL (p = 0.006). Similar rates of discontinuation
CVD prevention protocol including low-dose combination pill (antihypertensive agent, aspirin, statin) with lifestyle modification and adherence strategies	Zou et al.[10]	Rural China	CVD/Secondary prevention	Prospective, non-randomized, single center study; pilot before RCT; 153 patients to intervention, no control	Subjects aged 40–74 years with a calculated 10-year CVD risk > = 20%; mean age 71; male 71%	Intervention and follow-up: 3 months	BP, medication adherence, self-reported adherence to smoking cessation and salt intake, appointment rates	Significantly higher rates of subjects taking CVD preventive drugs (73% vs 84%, p = 0.000) and reduction in smoking rates (38% vs 35%, p = 0.007). No changes in salt intake or measured BP
**Mobile health**								
Education, counseling, and medication adjustment by nurses via phone calls	Ferrante et al.[36]	Argentina	Congestive heart failure	Prospective, multicenter, randomized, controlled trial; 760 patients to intervention, 758 usual care	Outpatients with stable CHF; mean age 65; male 71%	Mean intervention and follow-up: 16 months	All-cause mortality and CHF hospitalization	Relative risk reduction in primary outcome of 20% (RR 0.8 [0.66,0.97], p = 0.026), mostly driven by lower CHF hospitalization. No effect on all-cause mortality. Intervention patients had better quality of life (QOL) scores and medication adherence
Education, counseling, and medication adjustment by nurses via phone calls	Ferrante et al.[37]	Argentina	Congestive heart failure	Prospective, multicenter, randomized, controlled trial; 760 patients to intervention, 758 usual care	Outpatients with stable CHF; mean age 65; male 71%	Intervention: 1 year. Follow-up: 4 years	All-cause mortality and CHF hospitalization	2% absolute risk reduction in composite outcome of mortality or CHF hospitalization at 3 years (RR 0.88 [0.77,1.00], p = 0.05), mostly driven by 7% absolute risk reduction in CHF hospitalization at 3 years (RR 0.72 [0.60,0.87], p = 0.0004). Intervention patient had significantly higher rates of medication adherence and higher QOL scores
Phone call from nurses	Nesari et al.[16]	Iran	Diabetes	Prospective, randomized, controlled trial; 30 subjects to intervention, 31 to control	Subjects with diabetes; mean age 51; male 20% in control and 37% in intervention groups	Intervention and follow-up: 3 months	HbA1c	1.87% absolute decrease in HbA1c in intervention group (p<0.001) compared to no change in control group. Intervention group also saw significantly higher diet, exercise, glucose monitoring adherence
Automated SMS messages	Ramachandran et al.[17]	India	Diabetes	Prospective, multicenter, randomized controlled trial; 271 subjects to intervention, 266 to control	Men with impaired glucose tolerance (IGT); mean age 45–46	Mean intervention and follow-up: 20.2 months	Progression to diabetes	9% absolute lower progression to diabetes (18% vs 27%, hazard ratio 0.64 [0.45,0.92], p = 0.015). Also improved dietary adherence (hazard ratio 0.48 [0.33,0.71])
Automated SMS messages	Shetty et al.[18]	India	Diabetes	Prospective, randomized, controlled trial; 110 subjects to intervention and 105 to control	Subjects with diabetes; mean age 50	Intervention and follow-up: 1 year	HbA1c, fasting plasma glucose, lipids	Significant improvement in fasting plasma glucose in intervention group (166 vs 185 mg/dL, p<0.002). But no significant difference in HbA1c
Automated SMS messages	Goodarzi et al.[19]	Iran	Diabetes	Prospective, randomized controlled trial; 43 subjects to intervention, 38 control	Subjects with Type 2 diabetes; mean age 51–56; male 21–24%	Intervention and follow-up: 3 months	Laboratory results and questionnaire	0.89% absolute decrease in HbA1c (p = 0.024). Also significant decreases in total cholesterol and microalbumin. Significant improvement in questionnaire on knowledge, attitude, practice, and self-efficacy
Automated SMS message reminders	Khonsari et al.[38]	Malaysia	Coronary artery disease	Prospective, open-label, single center, randomized, controlled trial; ACS patients at tertiary teaching hospital; 31 patients to intervention and 31 to control	Participants admitted for ACS; mean age 58; male 86%	Intervention and follow-up: 2 months	Adherence to cardiac medications	Higher medication adherence rate (64.5% vs 12.9%, p<0.001). Intervention group trended towards lower hospital readmission rates (0% vs 12.9%, p = 0.056)
Phone call	Ortega et al.[39]	Brazil	Hypertension	Prospective, randomized, controlled trial; university hospital; 108 subjects to phone call, 246 to usual care	Subjects with hypertension; mean age 52–54; male 33–34%	Intervention and follow-up: 12 months	Medication adherence	3% absolute decrease in medication discontinuation rate (9.3% vs 12.2%, p<0.009). No difference in BP control
Automated phone calls and home BP monitors. Email alerts to providers	Piette et al.[40]	Honduras/Mexico	Hypertension	Prospective, randomized, controlled trial; primary care clinics; 99 subjects to intervention, 101 to control	Subjects with uncontrolled hypertension; mean age 58; male 33%	Intervention and follow-up: 6 weeks	BP	No significant effect on SBP. But in sub-group analysis, 8.8mm Hg SBP reduction in low literacy group (p = 0.002)
Two interventions: 1) 1-month free treatment (“incentive group”) or 2) reminder letters after missed follow-up (“letter group”)	Labhardt et al.[41]	Cameroon	CVD/Secondary prevention	Prospective, cluster-randomized, three-arm, open label study; nurse-led facilities; 11 facilities to control, 11 to “incentive group”, 11 to “letter group”	Subjects with hypertension or diabetes; 92 patients in control, 57 in “incentive group”, 80 in “letter group”; mean age 58–60; male 35–36%	Intervention and follow-up: 1 year	Retention rate at one year	Increased patient retention rates in both groups (60%/65% vs 29%, p<0.001). No difference between the two interventions. No significant differences in BP or fasting blood glucose
Telephone based peer support	Chan et al.[20]	Hong Kong	Diabetes	Prospective, multicenter, randomized, clinical trial; Hospital based diabetes centers; 316 subjects to control, 312 to intervention	Subjects with Type 2 diabetes; mean age 55; male 57%	Mean intervention and follow-up: 414 days	HbA1c, BP, total cholesterol, LDL	No significant changes in quality measures
Telephone based peer support	Rotheram-Borus et al.[21]	South Africa	Diabetes	Prospective, single center, non randomized, clinical trial; 22 female subjects to intervention	Subjects with diabetes; mean age 53; all female	Intervention: 3 months. Follow-up: 6 months	Blood glucose, body mass index, coping and social support	No significant improvements in clinical measures. In fact, blood glucose and diastolic BP increased. Social support and coping abilities increased
**Task shifting**								
Counseling by pharmacist, telephone reminders	Ramanath et al.[11]	India	Hypertension	Prospective, randomized, controlled trial; 26 subjects to intervention, 26 to control	Subjects with hypertension; male 62–81%	Intervention and follow-up: 1 month	BP, self-reported medication adherence	No significant effect on BP. Increased self-reported medication adherence
Nurse-led clinic and subsidized hypertensive medications	Kengne et al.[12]	Sub-Saharan Africa	Hypertension	Prospective, non-randomized, no control study; 5 urban and rural clinics; 454 subjects to intervention who served as own control	Subjects with hypertension; mean age 53–58; male 41–55%	Median intervention and follow-up: 6 months	BP	11.7mm Hg decrease in SBP and 7.8mm Hg decrease in DBP (p<0.001)
Pharmacist-led hypertension clinic	Erhun et al.[13]	Nigeria	Hypertension	Prospective, randomized cohort trial; state comprehensive health center; 51 subjects to intervention, no control	Subjects with uncontrolled hypertension; mean age 61; male 29%	Intervention and follow-up: 1 year	BP	Mean BP decreased from 168/103 at enrollment to 126/80 at fifth visit
Home visits	Adeyemo et al.[14]	Nigeria	Hypertension	Prospective, randomized controlled trial; rural and urban populations; 280 subjects to intervention, 264 to control	Subjects with hypertension; mean age 63; male 51–53%	Intervention and follow-up: 6 months	Medication adherence via pill counting or urine test	No difference in adherence
Family based home health education for patients and training of general practitioners (GP)	Jafar et al.[15]	Pakistan	Hypertension	Prospective, cluster-randomized, controlled trial; geographic census-based clusters; 629 subjects to intervention, 640 to control	Subjects with hypertension; mean age 54; male 37%	Intervention and follow-up: 2 years	SBP	10.8mm Hg absolute decrease in SBP (vs 5.8mm Hg, adjusted ratio 2.2 [1.3,3.6], p<0.001)
Pharmaceutical care program	Obreli-Neto et al.[42]	Brazil	Diabetes	Prospective, randomized, single center, controlled trial; primary health care clinic; 97 subjects to intervention, 97 to control	Elderly diabetic and hypertensive patients; mean age 65; male 37–38%	Intervention and follow-up: 36 months	Medication adherence and clinical outcomes (HbA1c, BP, lipids)	60% absolute increase in HbA1c<7 (p<0.001) and in BP<140/90 (p<0.001). 33% absolute increase in medication adherence (83.5% vs 52.6%, p<0.001).
**Guideline implementation**								
Training GPs in hypertension management	Qureshi et al.[43]	Pakistan	Hypertension	Prospective, cluster randomized, controlled trial; communities in Karachi; 100 subjects to intervention, 100 to control	Subjects with hypertension; mean age 55; male 38%	Intervention and follow-up: 6 weeks	Medication adherence	16% absolute increase in patient medication adherence (48.1% vs 32.4%, p = 0.048)
Clinical decision support system	Anchala et al.[44]	India	Hypertension	Prospective, cluster randomized, controlled trial; 8 primary health clusters in each arm; 845 subjects in intervention, 793 in control	Subjects with hypertension; mean age 54; male 49–52%	Intervention and follow-up: 12 months	SBP, cost effectiveness	6.59mm Hg absolute decrease in SBP (95% CI [1.42,12.2], p = 0.021). Cost effectiveness ratio was $96.01 per mm of SBP reduction in intervention arm and $36.57 in control arm
Patient education and medication (hydrochlorothiazide at 4 months if medium CVD risk)	Mendis et al.[45]	China and Nigeria	Hypertension	Prospective, cluster randomized, controlled trial; primary care clinics in China and Nigeria; 1191 subjects to intervention, 1206 to control	Subjects with hypertension; mean age 53–55; male 41–48%	Intervention and follow-up: 12 months	BP	Significant SBP decreases in both countries. China: SBP (13.3 vs 9.4mm Hg, p<0.0001), DBP (6.1 vs 4.5mm Hg, p = 0.0005). Nigeria: SBP (11.0 vs 6.6mm Hg, p = 0.0002), DBP (5.4 vs 2.0mm Hg, p<0.0001)
Education of GP regarding management guidelines including meetings, reminders, medical record summary, and patient result cards	Reutens et al.[46]	Asia	Diabetes	Prospective, cluster-randomized, multinational, controlled trial; 50 subjects to intervention, 49 to control	Asia-Pacific GPs; mean age 44; male 50–57%	Intervention and follow-up: 12 months	Patient HbA1c, BP, lipids	No significant difference in HbA1c or other glycemic indices
Incorporated chart with guidelines for diabetes and hypertension in each chart for providers	Steyn et al.[47]	South Africa	Diabetes	Prospective, multicenter, randomized controlled trial; public sector community health centers; 9 centers to intervention, 9 to control	Subjects with diabetes or hypertension; 690 intervention, 686 control; mean age 58–61; male 72–83%	Intervention and follow-up: 1 year	BP, HbA1c	No effect. Less than 60% of guideline forms used
**Patient education**								
Adherence therapy	Alhalaiqa et al.[48]	Jordan	Hypertension	Prospective, randomized controlled trial; outpatients clinics in three large government-run hospitals; 68 subjects to intervention, 68 to control	Non-compliant hypertension patients; mean age 53; male 37–56%	Intervention: 7 weeks. Follow-up: 11 weeks	BP, medication adherence (via pill counting)	SBP decreased by 21.6mm Hg (adjusted p<0.01) and DBP by 12.8mm Hg (adjusted p<0.01). 26.4% higher absolute increase in medication adherence ([23.4, 29.4], p<0.01)
Patient education and medication (hydrochlorothiazide at 4 months if medium CVD risk)	Mendis et al.[45]	China and Nigeria	Hypertension	Prospective, cluster randomized, controlled trial; primary care clinics in China and Nigeria; 1191 subjects to intervention, 1206 to control	Subjects with hypertension; mean age 53–55; male 41–48%	Intervention and follow-up: 12 months	BP	Significant SBP decreases in both countries. China: SBP (13.3 vs 9.4mm Hg, p<0.0001), DBP (6.1 vs 4.5mm Hg, p = 0.0005). Nigeria: SBP (11.0 vs 6.6mm Hg, p = 0.0002), DBP (5.4 vs 2.0mm Hg, p<0.0001)
Partnership meeting ("Motivation, Readying, Involvement, and Evaluation")	Mohammadi et al.[49]	Iran	Hypertension	Prospective, cluster-randomized, controlled trial; rural health center; 75 subjects to intervention, 75 to control	Subjects with hypertension	Intervention and follow-up: 1 year	BP	32% absolute increase in well-controlled BP (<140/90) (33.3% vs 1.3%, p<0.005)
Diary check list	Ahmadipour et al.[50]	Iran	Diabetes	Prospective, single center, randomized trial; 30 subjects in diary group and 57 in control group	Subjects with Type 2 diabetes; mean 48–52; male 10–18%	Intervention and follow-up: 12 weeks	HbA1c, medication adherence rate	Higher adherence rate for check list group than pocket group (96.7% vs 55.2%, p = 0.02). No significant difference in HbA1c
Monthly diabetes education program	Tan et al.[51]	Malaysia	Diabetes	Prospective, single-blind, randomized, controlled trial; 82 subjects to intervention, 82 to control	Subjects with poorly controlled diabetes; mean age 54; male 38–39%	Intervention and follow-up: 12 weeks	HbA1c, SMBG (self measured blood glucose), questionnaire measured knowledge, self-reported medication adherence	0.9% absolute decrease in HbA1c (8.75% vs 9.67%, p<0.001). Also increased SMBG, medication adherence, and knowledge
Collaborative practice intervention	Arevian et al.[52]	Lebanon	Diabetes	Prospective, single center study	Patients with diagnosed diabetes	Implementation and follow-up: 1 year	Documentation, patient recruitment, continuity of care, glycemic control, direct cost of care	Increased documentation, patient recruitment, continuity of care, and glycemic control. Direct cost of care less than university private clinic
Disease management programs: small group education, exercise training, regular nursing consultation	Andryukhin et al.[53]	Russia	Congestive heart failure	Prospective, randomized, controlled trial; 44 patients to intervention, 41 to control	Subjects with diastolic CHF; mean age 67; male 27–34%	Intervention: 6 months. Follow-up: 18 months	Physical parameters, symptoms, echocardiogram, mortality, admission	Improved 6-minute walk test (68% vs 20%, p<0.001) and left ventricular end-diastolic volume index (70% vs 46%, p = 0.032). Also improved lipids (total cholesterol, LDL) and QOL scores. No difference in mortality or admissions
Disease management program	Bocchi et al.[54]	Brazil	Congestive heart failure	Prospective, single center, randomized, controlled trial; 223 intervention, 117 control	Subjects with chronic CHF; mean age 50–52; male 64–71%	Mean intervention and follow-up: 2.47 years	Composite primary outcome (death and unplanned hospitalization)	Reduction in composite outcome (hazard ratio 0.64 [0.43,0.88], p = 0.008), driven by reduction in hospitalization. Lower total hospital days and need for emergency visits

### System level-acute phase CVD

Three studies examined interventions in the system level-acute phase category[22–24] (Table 2). Alexander et al. reported on a project being launched in a rural region within Tamil Nadu state, India that plans to implement a “hub-and-spoke” model using existing health care facility and ambulance transportation resources to improve the acute ST-elevation myocardial infarction care delivery system. Another multi-component intervention study, the Kerala ACS program included community-based health education programs that promoted self-detection of acute coronary disease symptoms, rapid self-referral for treatment, and timely self-administration of aspirin [22]. The investigators concluded that improved patient awareness of ACS symptoms and treatments contributed to reduced time-to-thrombolysis achieved by the multi-component intervention. We found no studies assessing the impact of improved geographical and temporal coverage of acute care services, including the impact of building more hospitals within under-served areas, expanding service hours to include more overnight capacity, or making revascularization more widely available within the health system.

### System level-chronic phase CVD

Most studies in the system level-chronic phase category studied expansion of health insurance coverage [25–32] (Table 3). Two studies evaluated the health impact of *Seguro Popular* insurance rolled out in 2002 as part of Mexico’s national universal health insurance plan. *Seguro Popular* covered approximately 50 million low-income people who previously had no formal health insurance–often because working family members participated in the informal economy [25]. Based on data gathered in Mexican national health and nutrition surveys during step-wise introduction of insurance, Bleich et al. found that compared with matched hypertensive adults without insurance, hypertensive *Seguro Popular* enrollees had 1.5-fold higher odds of receiving hypertension treatment and 1.4-fold higher odds of having controlled blood pressure (BP) [25]. A similar study of low-income diabetic patients found those with *Seguro Popular* insurance were more likely to receive regular blood glucose control monitoring and maintain adequate glucose control compared to their matched, uninsured counterparts [26].

In rural Nigeria, hypertensive patients living in a district where community-based health insurance was available had significantly lower SBP and diastolic blood pressure (DBP) not observed in the control group without insurance [27]. In rural China, hypertensive patients receiving subsidies to defer medication costs had a 9% absolute increase in medication adherence and significantly lower annual out-of-pocket medical costs [28].

### Patient/provider level-acute phase CVD

Most studies in the patient/provider level–acute phase category studied ACS clinical pathways[3, 5, 22, 33] (Table 4). Berwanger et al. randomized large urban hospitals in Brazil to either a multifaceted quality improvement program with educational materials, reminders, treatment algorithms and training visits or to usual care. The intervention group had 2.64 higher odds (95% confidence interval 1.28 to 5.45) of receiving evidence-based ACS treatment within the first 24 hours following symptom onset[5]. However, there was no difference observed in 30-day mortality or rate of in-hospital repeat cardiovascular events.

In urban China, Du et al. randomized hospitals to implementation of a United States guideline-based ACS pathway, along with interval clinical performance audits and feedback throughout the intervention period and compared the results of these interventions to hospitals continuing usual care practices. Intervention hospitals had higher rates of discharge on recommended therapies compared to usual care hospitals, but no difference was observed in other indicators, including reperfusion within 12 hours of symptom onset in ST-elevation myocardial infarction cases, door-to-needle time, door-to-balloon time, or proportion of high-risk patients undergoing angiography[3]. Similar to the Berwanger et al. study, there was no significant difference in mortality or cardiovascular morbidity events. In a third study, Prabhakaran et al. enrolled 34 hospitals in the Kerala region of India to serve as their own controls in a pre- and post-intervention comparison design. After the multi-faceted quality improvement intervention, there was a significant median reduction of 54 minutes in time-to-thrombolysis from 193.0 to 139.0 minutes and a significant increase in utilization of evidence-based medications[22].

### Patient/provider level-chronic phase CVD

The majority of selected studies (33/49) were in the patient/provider level-chronic phase category [8–21, 35–54] (Table 5). CVD patients are often prescribed several medications for primary or secondary prevention, making *medication adherence* a key challenge to delivering quality CVD clinical care worldwide. Thus most interventions were related to chronic medication adherence. Types of medication adherence interventions included use of fixed-dose combination pills, “mobile” health care, task shifting, clinical guideline implementation, and patient education.

#### Improving patients’ medication adherence through combination pills

Thom et al. randomized high-risk CVD patients in India (one of four countries studied) to receive combination pills or the usual, multiple-pill therapy. After a mean follow-up of about 15 months, Indian participants randomized to combination pills had a 25% higher absolute adherence compared to those randomized to usual care (89.1% vs 63.6%, unadjusted relative risk 1.4, 95% confidence interval 1.30 to 1.51, p<0.001) and small but significant reductions in both SBP and low-density lipoprotein (LDL) compared with participants randomized to multiple-pill treatments [8]. In another study, Yusuf et al. also found small but significant improvement in adherence among high CVD risk Indian subjects randomized to a combination pill containing multiple BP medications, statins, and aspirin [9]. A follow-up study showed that high-dose combination pills improved BP and lipid control in high CVD risk Indian subjects compared to low-dose combination pills, while both formulations had similar rates of tolerability [35]. Zou et al. found that high CVD risk rural Chinese participants achieved an 11% higher absolute adherence rate after they were offered combination pills in a pre- and post-intervention comparison design (84% vs 73%, p = 0.000)[10]

#### Improving patients’ medication adherence through mobile health interventions

Twelve studies investigated the role of mobile health in prevention and management of CHF, diabetes, hypertension, and coronary artery disease in LMICs [16–21, 36–39, 41]. Chronic CHF patients in Argentina were randomized to a telemedicine intervention in which nurses called patients to adjust medications depending on their symptoms and provided counseling and education or to usual care. Patients in the intervention arm had a 20% relative risk reduction (RR = 0.80, 95% confidence interval 0.66 to 0.97, p = 0.026) in the primary composite outcome of all-cause mortality and CHF hospitalizations, driven mostly by the latter outcome component[36]. A follow-up study showed that even after three years, CHF hospitalizations, medication adherence rates, and quality of life scores had improved significantly more in the intervention group [37]. Similarly, Nesari et al. showed that Iranian diabetic patients randomized to a nurse telephone follow-up intervention had significant improvements in their HbA1c and adherence to healthy lifestyle changes compared to those randomized to usual care [16].

Four studies evaluated the effects of automated phone texts on diabetes and coronary artery disease prevention. Ramachandran et al. and Shetty et al. found that automated SMS (short message service) messages led to improved glycemic control in Indians with pre-diabetes and diabetes[17, 18]. Goodarzi et al. showed an absolute decrease in HbA1c in Iranian diabetic subjects who received automated SMS messages[19]. Khonsari et al. revealed that automated SMS messages led to higher medication adherence rates in Malaysian patients after hospital discharge following ACS [38]. It is important to note that all four studies only included participants with regular access to mobile phones with text receiving capabilities.

#### Task shifting to increase patients’ medication adherence

Task shifting refers to the redistribution of tasks among health workforce teams, often from a few, highly trained health providers to a larger contingent of providers with less formal health care training[55]. Our review identified six studies evaluating task shifting for improving patient adherence to prescribed medications [11–15, 42]. Some studies coupled task shifting with increased access to affordable or free medications [12, 13] or family-based home health education and supplemental training of general practitioners [15].

Five task-shifting studies targeted hypertensive patients[11–15]. Regardless of the approach, intensified, team-based care led to improved hypertension control. Kengne et al. carried out a large trial of hypertensive participants enrolled in a nurse-led clinic in Cameroon. Erhun et al. evaluated the role of pharmacist-led clinics in Nigerian patients with hypertension. Adeyemo et al. randomized Nigerian participants with hypertension to clinic-based care with home visits or to clinic-based care only. Jafar et al. was a cluster-randomized, controlled trial of two different interventions, home health education by health aides and training of general practitioners, in a population of Pakistani patients with hypertension.

Two studies focused on task shifting interventions in diabetic patients [16, 42]. Nesari et al., as mentioned above, showed that having nurses call patients regularly to reinforce lifestyle changes and adjust medication doses led to a significant decrease in HbA1c. The intervention group also showed increased rates of adherence to lifestyle changes and glucose monitoring.

#### Guideline implementation and provider education leading to improved patient outcomes

Health care provider education and guideline implementation have the potential to standardize, improve, and sustain quality of care for vascular and other conditions in LMICs. Studies of the impact of physician education and guideline dissemination yielded mixed results[43–47]. Qureshi et al. showed that physician education through workshops and guideline dissemination led to significant improvements in quality of hypertension management. Anchala et al. revealed that providing physicians with a clinical decision support system to undertake guideline-based hypertension management led to significant reductions in SBP. Mendis et al. showed that dissemination of an evidence-based cardiovascular risk management package led to significant reductions in SBP in both Chinese and Nigerian primary care clinics compared to usual care (China: 13.3 vs 9.4mm Hg, p<0.0001; Nigeria: 11.0 vs 6.6mm Hg, p = 0.0002) [45]. However, Reutens et al. and Steyn et al. showed conflicting results and highlighted the fact that guideline dissemination alone did not lead to implementation or improve health outcomes in South African clinics.

#### Educating patients to enhance health outcomes

The review found eight studies that evaluated disease management education among patients with hypertension, diabetes, and CHF. [48–54]. Alhalaiqa et al. and Mohammadi et al. showed that hypertensive patients in Jordan and Iran, respectively, who were randomized to medication adherence education and motivational group meetings had improved SBP and medication adherence compared to those participants in the control groups. Likewise, Tan et al. revealed that Malaysian patients with poorly controlled diabetes who were randomized to monthly diabetes education programs resulted in 0.9% absolute reduction in HbA1c (8.75 vs 9.67%, p<0.001), changes not observed in the control group [51]. Bocchi et al. reported Brazilian CHF patients randomized to disease management programs had a delay in median follow-up time to first hospitalization by 2.2 years (4.21 vs 2.06 years, p = 0.0041) compared to those in the control group, though mortality was similar in both groups [54]. Conversely, Andryukhin et al. did not find that disease management led to decreased hospital admissions or mortality, but did lead to improved functional status and quality of life.

### Discussion

**Fig 3:**
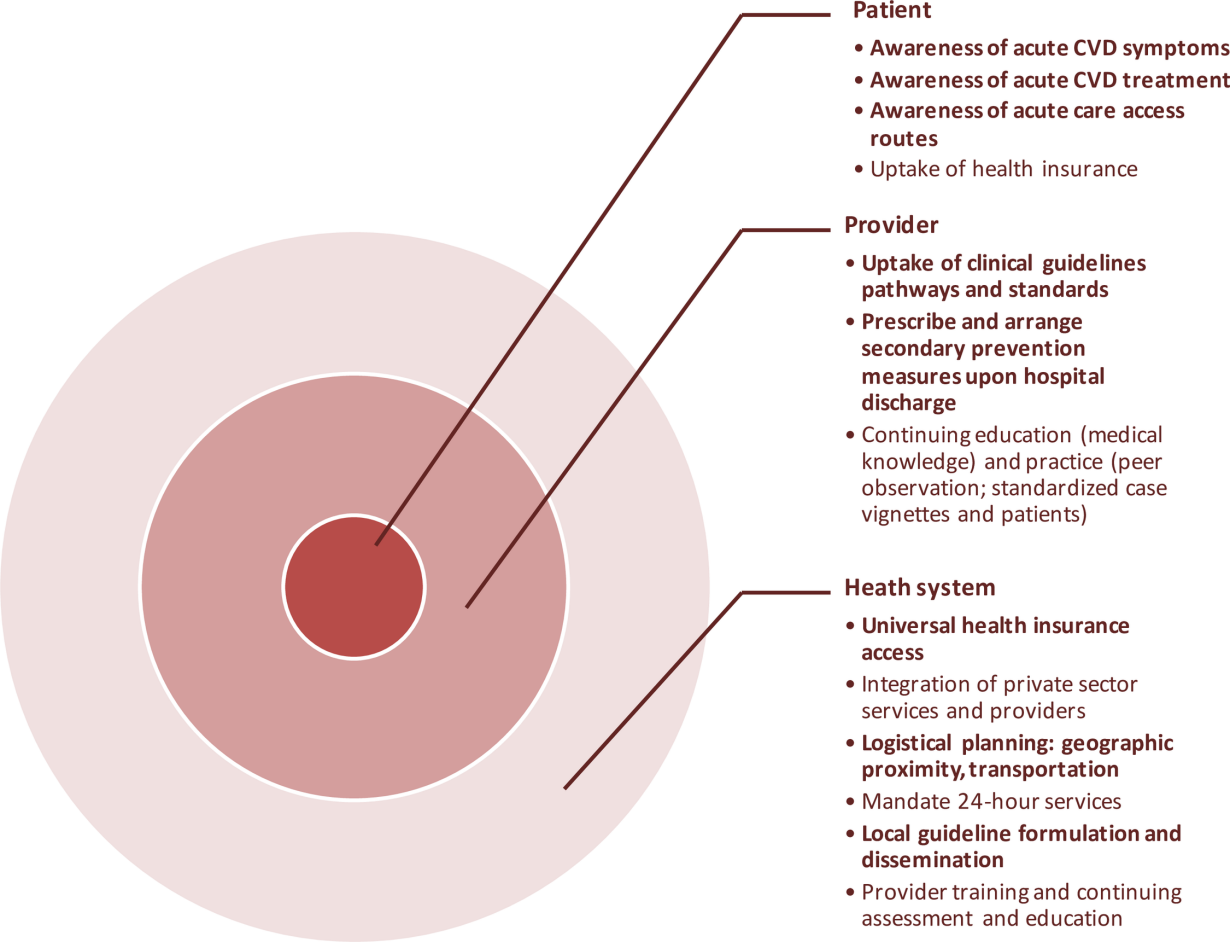
Examples of acute phase CVD quality improvement interventions identified in the DCP3 systematic review. Types of interventions targeting three levels of acute phase CVD prevention and management. Bullets in bold type are supported by evidence from the review. Bullets without bold type indicate that no supporting evidence was found in the review and are potential areas for further research.

**Fig 4:**
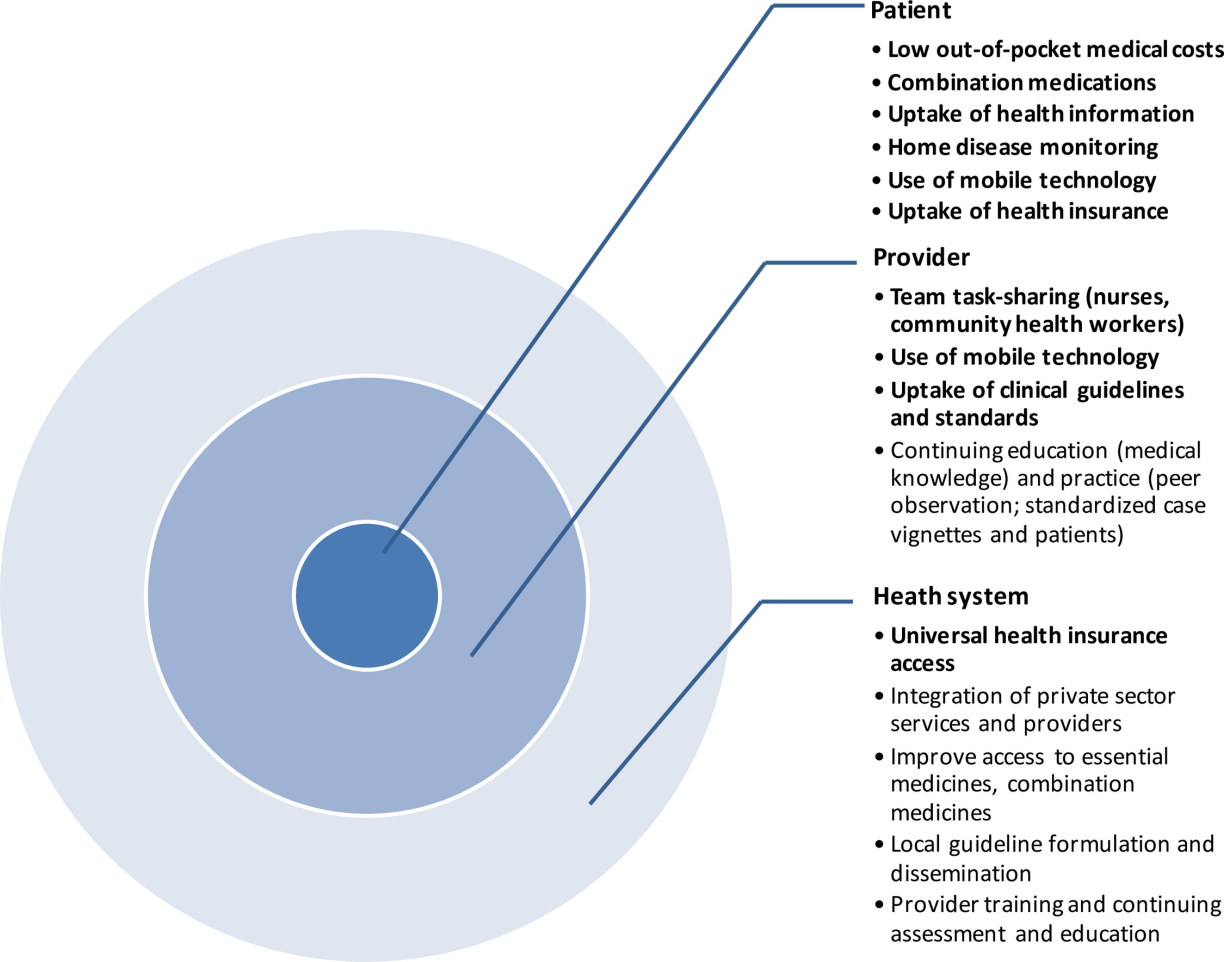
Examples of chronic phase CVD quality improvement interventions identified in the DCP3 systematic review. Types of interventions targeting three levels of chronic phase CVD prevention and management. Bullets in bold are supported by evidence from the review. Bullet without bold type indicate that no supporting evidence was found in the review and are potential areas for further research.

Our systematic review identified 49 original studies of CVD care quality improvement in LMICs. Taken together, the evidence suggests that quality improvement programs have the potential to improve CVD care in LMICs, whether for treating acute events or in long-term prevention and chronic disease management. Not all approaches reviewed were consistently effective, and we did not find evidence supporting all the approaches we intended to assess *a priori* (Figs 3 and 4).

### System level-acute phase CVD

Our systematic review found limited evidence in the acute phase-system level category. Perhaps poor underlying infrastructure in low resource settings presents daunting challenges to re-organize complex health delivery systems [56]. Just as likely, governments, non-governmental, and private sector organizations often introduce system improvements without rigorous systematic study. Therefore, health effects of system level changes may go unmeasured or unreported. Conducting randomized comparison studies in low- and middle-income settings may be limited by lack of research capacity, perception of causing unwanted delay in care delivery, “contamination” between intervention and control sites, and ethical concerns. The lack of evidence is unfortunate because timely intervention can dramatically improve acute CVD outcomes, and delays may result in unnecessary death or disability. Awareness, acceptability, affordability, and availability are all common barriers that can delay treatment of acute events. Community education and system-wide infrastructure, logistics, and human resource planning can overcome barriers to timely and appropriate care for acute CVD. Integration of private sector ambulance companies, hospitals, and providers is an important part of achieving improved quality of acute CVD care in LMICs, where the private sector provides a large proportion of health care and may be in the position to efficiently fill gaps in public health care systems [57].

### System level-chronic phase CVD

Unfortunately, many CVD patients in LMICs remain untreated or incompletely treated with standard oral medications for secondary prevention [58]. Our review suggests that system level quality improvement efforts can lead to measurable improvements in health status in LMIC patients with chronic CVDs. Implementation of universal health insurance led to significant improvements in chronic disease management in Latin America, Africa, and Asia [25–28]. Our review captured no studies of the impact of “essential medicines” designations and price controls or pharmaceutical market regulation on the quality of CVD clinical care.

In sub-Saharan Africa, the substantial infrastructure investment that turned the tide of the HIV epidemic is now being leveraged for chronic non-communicable disease management[59]. Groups like the Kenya-based Academic Model Providing Access to Healthcare (AMPATH) have leveraged this health system platform to build a comprehensive, multi-level approach to quality improvement in order to improve cardiovascular care in the communities they serve[60, 61]. Results of this multi-level intervention are expected soon.

System level interventions have been systematically evaluated through pre- and post-intervention “natural experiment” comparisons, observational comparisons of intervention participants and matched usual care participants, or comparisons of geographic areas randomly assigned to receive or not receive the intervention. Stepped-wedge trials introduce interventions to couple step-wise active and systematic program implementation with evaluation [62]. As in the Mexican *Seguro Popular* studies, repeated population-based surveys (national census or health surveys) can be leveraged to measure changes in chronic CVD risk factors and outcomes. Quality improvement studies will be most feasible where key outcomes are part of, or added to, ongoing surveys.

### Patient/provider–acute phase CVD

Adopting clinical practice guidelines from high-income countries to LMICs appears to be a great opportunity both for implementing quality improvement standards and benchmarking significant practice and outcome improvements. The ACS and stroke quality improvement studies identified in our review showed some clinical process measure improvements, but like high-income region studies, quality did not consistently improve for all pre-specified outcomes [3, 5, 34]. Success of acute phase CVD clinical pathway interventions likely depends on the support of health care providers and administrators, and tailoring to the specific context of the participating health care system (i.e., availability of treatments and financial protection for patients) [63]. Lessons learned from these programs may be helpful for the design of future acute, patient/provider level CVD quality improvement studies. Despite their limitations, these ambitious studies demonstrated that large-scale and complex quality improvement programs can be implemented in LMIC hospitals. Absent from our review were studies measuring the impact of physician education on diagnostic accuracy or clinical decision-making related to acute cardiovascular disorders.

### Patient/provider–chronic phase CVD

Adherence to life saving medications and lifestyle changes is suboptimal world-wide, regardless of country income level [58]. The most consistent general finding among selected LMIC studies was that increased *intensity* of care improved chronic disease and risk factor outcomes, regardless of how or through whom it was delivered. While care intensification inevitably requires up-front investment, this investment may be offset by improved downstream CVD outcomes, health gains, and cost-savings. A modeling study by Gaziano et al. projected that despite added costs of hiring community health workers to manage hypertension in South Africa, the intervention would be cost-effective due to health gains and cost savings from prevented hospital admissions and chronic disease complications [64]. The trials reviewed also suggest that combination pills have the potential to improve medication adherence and improve risk factor control in high-risk patients without necessarily adding extra costs. Efforts to efficiently manufacture and gain regulatory approval for combination medications are underway [65].

Rapidly increasing access to mobile telecommunications technology may also be a promising tool for chronic disease management. There are now almost seven billion mobile phone users world-wide with more than three-quarters of these users living in LMICs [66]. A 2012 Cochrane review cautiously concluded that there was some limited evidence that mobile phone messaging may be beneficial in self-management of chronic diseases [67]. This was based on evidence from four studies, all performed within high-income countries. We found a number of studies in LMICs that successfully leveraged the ubiquity and low cost of mobile technology to improve CHF, hypertension, and diabetes care outcomes.

We found that efforts to improve quality care through physician education and guideline dissemination yielded inconsistent results [43, 44, 46, 47]. Guideline dissemination did not lead to actual implementation in all cases. Gaining buy-in from providers appears to be important for success. Allocating time for education and feedback and strategically inserting guideline information into the flow of clinical practice may increase the chance that guidelines are actually implemented.

### Limitations

**Fig 5:**
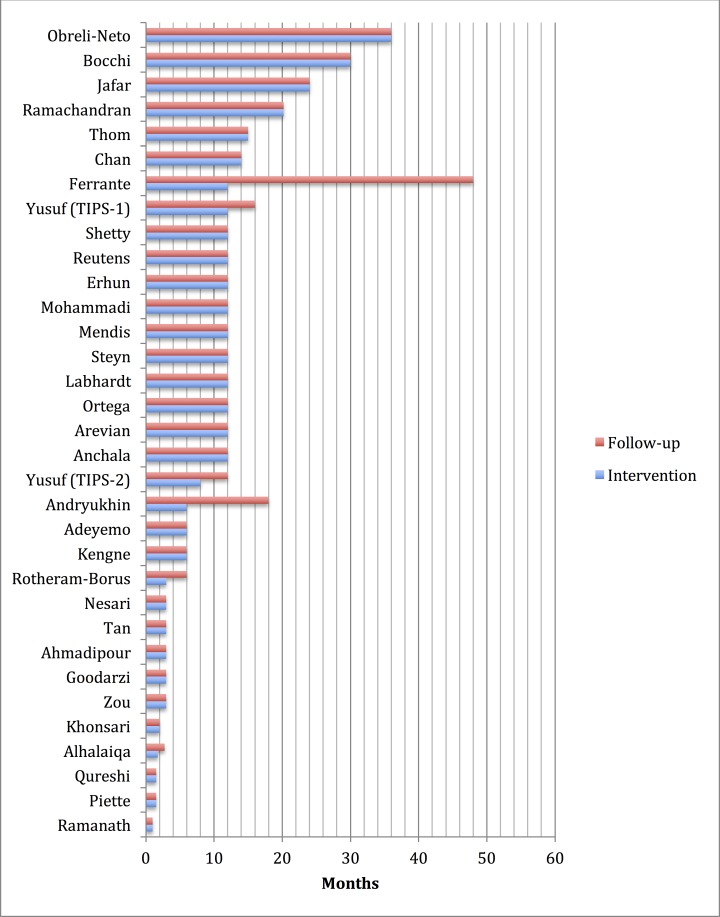
Duration of chronic cardiovascular disease or risk factor management studies identified in the DCP3 systematic review.

Our review and the studies we reviewed had limitations, so our results should be interpreted with caution. Due to the diversity of interventions and conditions, we were unable to summarize effect sizes in a meta-analysis. It is possible that most of our included studies were published because of “positive” results—due to the heterogeneity in interventions and targets, we were not able to formally evaluate the results of our review for evidence of publication bias. Also important is that most study durations were relatively short term (<12 months) and sustaining intervention effects may be a big challenge when these programs are implemented in real clinical settings (Fig 5). As in high-income countries, most investigators studied clinical process measures, and did not report on hard clinical outcomes, which may be more vulnerable to “gaming” the system to produce only nominal improvements and other unintended consequences when these interventions are introduced into routine practice.

## Conclusions

We identified good evidence that short-term quality improvement interventions improved the quality of cardiovascular care in low- and middle-income countries, especially for chronic conditions delivered at the patient/provider level. Most studies were short-term. At the patient/provider level, intensified, team-based care generally led to improved medication adherence and hypertension control. At the system level, studies provided evidence that introduction of universal health insurance coverage improved hypertension and diabetes control. Studies of system and patient/provider level ACS quality improvement interventions yielded inconclusive results.

What is needed now is more evidence that quality improvement can be sustained in the long term while remaining effective and affordable. Collecting data elements common to implementation research such as acceptability, sustainability, local context, and affordability and collecting and assessing qualitative and process data will help ensure that both positive and negative studies will guide implementation and future research[68].

The majority of CVD patients now live in LMICs, and demographic trends virtually guarantee that the number and proportion will grow in coming decades. In order to ensure that each of these patients receives high quality preventive and acute care, we will need to draw on promising research on clinical quality improvement in order to make the most of the resources directly at hand.

## Supporting Information

S1 Fig PRISMA Checklist. (TIF)

S1 Table Search terms on PubMed (June 9, 2014). (TIF)

S2 Table Search terms on EMBASE (June 9, 2014). (TIF)

## References

[1] Roth GA, Huffman MD, Moran AE, Feigin V, Mensah GA, Naghavi M, et al Global and Regional Patterns in Cardiovascular Mortality From 1990 to 2013. Circulation. 2015;132(17):1667–78. 10.1161/circulationaha.114.008720 26503749

[2] Institute of Medicine Committee to Design a Strategy for Quality R, Assurance in M. In: Lohr KN, editor. Medicare: A Strategy for Quality Assurance: Volume 1 Washington (DC): National Academies Press (US) Copyright (c) 1990 by the National Academy of Sciences.; 1990.

[3] Du X, Gao R, Turnbull F, Wu Y, Rong Y, Lo S, et al Hospital Quality Improvement Initiative for Patients With Acute Coronary Syndromes in China: A Cluster Randomized, Controlled Trial. Circulation: Cardiovascular Quality and Outcomes. 2014;7(2):217–26. 10.1161/circoutcomes.113.000526 24619325

[4] Glickman SW, Ou F, DeLong ER, et al PAy for performance, quality of care, and outcomes in acute myocardial infarction. Jama. 2007;297(21):2373–80. 10.1001/jama.297.21.2373 17551130

[5] Berwanger O, Guimarães HP, Laranjeira LN, Cavalcanti AB, Kodama AA, Zazula AD, et al Effect of a Multifaceted Intervention on Use of Evidence-Based Therapies in Patients With Acute Coronary Syndromes in Brazil. Jama. 2012;307(19). 10.1001/jama.2012.413 22665103

[6] Galarraga O. Diabetes treatment and control: the effect of public health insurance for the poor in Mexico. Bulletin of the World Health Organization. 2009;87(7):512–9. 10.2471/blt.08.053256 19649365 PMC2704037

[7] Walker RW, Jusabani A, Aris E, Gray WK, Unwin N, Swai M, et al Stroke risk factors in an incident population in urban and rural Tanzania: a prospective, community-based, case-control study. The Lancet Global Health. 2013;1(5):e282–e8. 10.1016/s2214-109x(13)70068-8 24748275 PMC3986030

[8] Thom S. Effects of a Fixed-Dose Combination Strategy on Adherence and Risk Factors in Patients With or at High Risk of CVD. Jama. 2013;310(9):918 10.1001/jama.2013.277064 24002278

[9] Yusuf S, Pais P, Afzal R, Xavier D, Teo K, Eikelboom J, et al Effects of a polypill (Polycap) on risk factors in middle-aged individuals without cardiovascular disease (TIPS): a phase II, double-blind, randomised trial. Lancet. 2009;373(9672):1341–51. Epub 2009/04/03. 10.1016/s0140-6736(09)60611-5 . 19339045

[10] Zou G, Wei X, Gong W, Yin J, Walley J, Yu Y, et al Evaluation of a systematic cardiovascular disease risk reduction strategy in primary healthcare: an exploratory study from Zhejiang, China. Journal of public health. 2014 Epub 2014/04/04. 10.1093/pubmed/fdu013 . 24696086

[11] Ramanath K, Balaji D, Nagakishore C, Kumar SM, Bhanuprakash M. A study on impact of clinical pharmacist interventions on medication adherence and quality of life in rural hypertensive patients. Journal of young pharmacists: JYP. 2012;4(2):95–100. Epub 2012/07/04. 10.4103/0975-1483.96623 22754261 PMC3385224

[12] Kengne AP, Awah PK, Fezeu LL, Sobngwi E, Mbanya J-C. Primary Health Care for Hypertension by Nurses in Rural and Urban Sub-Saharan Africa. The Journal of Clinical Hypertension. 2009;11(10):564–72. 10.1111/j.1751-7176.2009.00165.x 19817937 PMC8673012

[13] Erhun WO, Agbani EO, Bolaji EE. Positive benefits of a pharmacist-managed hypertension clinic in Nigeria. Public health. 2005;119(9):792–8. Epub 2005/07/02. 10.1016/j.puhe.2004.11.009 . 15990127

[14] Adeyemo A, Tayo BO, Luke A, Ogedegbe O, Durazo-Arvizu R, Cooper RS. The Nigerian antihypertensive adherence trial: a community-based randomized trial. Journal of hypertension. 2013;31(1):201–7. Epub 2012/11/10. 10.1097/HJH.0b013e32835b0842 23137954 PMC3530610

[15] Jafar TH, Hatcher J, Poulter N, Islam M, Hashmi S, Qadri Z, et al Community-based interventions to promote blood pressure control in a developing country: a cluster randomized trial. Annals of internal medicine. 2009;151(9):593–601. Epub 2009/11/04. 10.7326/0003-4819-151-9-200911030-00004 . 19884620

[16] Nesari M, Zakerimoghadam M, Rajab A, Bassampour S, Faghihzadeh S. Effect of telephone follow-up on adherence to a diabetes therapeutic regimen. Japan journal of nursing science: JJNS. 2010;7(2):121–8. Epub 2010/11/26. 10.1111/j.1742-7924.2010.00146.x . 21092015

[17] Ramachandran A, Snehalatha C, Ram J, Selvam S, Simon M, Nanditha A, et al Effectiveness of mobile phone messaging in prevention of type 2 diabetes by lifestyle modification in men in India: a prospective, parallel-group, randomised controlled trial. The Lancet Diabetes & Endocrinology. 2013;1(3):191–8. 10.1016/s2213-8587(13)70067-6 24622367

[18] Shetty AS, Chamukuttan S, Nanditha A, Raj RK, Ramachandran A. Reinforcement of adherence to prescription recommendations in Asian Indian diabetes patients using short message service (SMS)—a pilot study. The Journal of the Association of Physicians of India. 2011;59:711–4. Epub 2012/05/24. . 22616337

[19] Goodarzi M, Ebrahimzadeh I, Rabi A, Saedipoor B, Jafarabadi MA. Impact of distance education via mobile phone text messaging on knowledge, attitude, practice and self efficacy of patients with type 2 diabetes mellitus in Iran. J Diabetes Metab Disord. 2012;11(1):10 Epub 2012/01/01. 10.1186/2251-6581-11-10 ; PubMed Central PMCID: PMCPmc3598175. 23497632 PMC3598175

[20] Chan JC, Sui Y, Oldenburg B, Zhang Y, Chung HH, Goggins W, et al Effects of telephone-based peer support in patients with type 2 diabetes mellitus receiving integrated care: a randomized clinical trial. JAMA internal medicine. 2014;174(6):972–81. Epub 2014/05/02. 10.1001/jamainternmed.2014.655 . 24781960

[21] Rotheram-Borus MJ, Tomlinson M, Gwegwe M, Comulada WS, Kaufman N, Keim M. Diabetes buddies: peer support through a mobile phone buddy system. The Diabetes educator. 2012;38(3):357–65. Epub 2012/05/02. 10.1177/0145721712444617 ; PubMed Central PMCID: PMCPmc4059372. 22546740 PMC4059372

[22] Prabhakaran D, Jeemon P, Mohanan PP, Govindan U, Geevar Z, Chaturvedi V, et al Management of acute coronary syndromes in secondary care settings in Kerala: impact of a quality improvement programme. The National medical journal of India. 2008;21(3):107–11. Epub 2008/11/14. . 19004139

[23] Alexander T, Victor SM, Mullasari AS, Veerasekar G, Subramaniam K, Nallamothu BK, et al Protocol for a prospective, controlled study of assertive and timely reperfusion for patients with ST-segment elevation myocardial infarction in Tamil Nadu: the TN-STEMI programme. BMJ open. 2013;3(12):e003850 Epub 2013/12/05. 10.1136/bmjopen-2013-003850 24302505 PMC3855601

[24] Nazzal NC, Campos TP, Corbalan HR, Lanas ZF, Bartolucci JJ, Sanhueza CP, et al [The impact of Chilean health reform in the management and mortality of ST elevation myocardial infarction (STEMI) in Chilean hospitals]. Revista medica de Chile. 2008;136(10):1231–9. Epub 2009/02/06. /S0034-98872008001000001. . 19194618

[25] Bleich SN, Cutler DM, Adams AS, Lozano R, Murray CJ. Impact of insurance and supply of health professionals on coverage of treatment for hypertension in Mexico: population based study. Bmj. 2007;335(7625):875 Epub 2007/10/24. 10.1136/bmj.39350.617616.BE 17954519 PMC2043407

[26] Sosa-Rubi SG, Galarraga O, Lopez-Ridaura R. Diabetes treatment and control: the effect of public health insurance for the poor in Mexico. Bulletin of the World Health Organization. 2009;87(7):512–9. 19649365 10.2471/BLT.08.053256 PMC2704037

[27] Hendriks ME, Wit FW, Akande TM, Kramer B, Osagbemi GK, Tanovic Z, et al Effect of health insurance and facility quality improvement on blood pressure in adults with hypertension in Nigeria: a population-based study. JAMA internal medicine. 2014;174(4):555–63. Epub 2014/02/19. 10.1001/jamainternmed.2013.14458 . 24534947

[28] Yu B, Zhang X, Wang G. Full coverage for hypertension drugs in rural communities in China. The American journal of managed care. 2013;19(1):e22–9. Epub 2013/02/06. . 23379776 PMC4538950

[29] Gaziano TA, Pandya A, Steyn K, Levitt N, Mollentze W, Joubert G, et al Comparative assessment of absolute cardiovascular disease risk characterization from non-laboratory-based risk assessment in South African populations. BMC medicine. 2013;11:170 Epub 2013/07/25. 10.1186/1741-7015-11-170 23880010 PMC3734109

[30] Beaton A, Okello E, Lwabi P, Mondo C, McCarter R, Sable C. Echocardiography screening for rheumatic heart disease in Ugandan schoolchildren. Circulation. 2012;125(25):3127–32. Epub 2012/05/26. 10.1161/CIRCULATIONAHA.112.092312 . 22626741

[31] Carapetis JR, Hardy M, Fakakovikaetau T, Taib R, Wilkinson L, Penny DJ, et al Evaluation of a screening protocol using auscultation and portable echocardiography to detect asymptomatic rheumatic heart disease in Tongan schoolchildren. Nature clinical practice Cardiovascular medicine. 2008;5(7):411–7. Epub 2008/04/10. 10.1038/ncpcardio1185 . 18398402

[32] Trujillo AJ, Vecino Ortiz AI, Ruiz Gomez F, Steinhardt LC. Health insurance doesn't seem to discourage prevention among diabetes patients in Colombia. Health affairs. 2010;29(12):2180–8. Epub 2010/12/08. 10.1377/hlthaff.2010.0463 . 21134918

[33] Flather MD, Babalis D, Booth J, Bardaji A, Machecourt J, Opolski G, et al Cluster-randomized trial to evaluate the effects of a quality improvement program on management of non-ST-elevation acute coronary syndromes: The European Quality Improvement Programme for Acute Coronary Syndromes (EQUIP-ACS). American heart journal. 2011;162(4):700–7 e1. Epub 2011/10/11. 10.1016/j.ahj.2011.07.027 . 21982663

[34] Peng B, Ni J, Anderson CS, Zhu Y, Wang Y, Pu C, et al Implementation of a structured guideline-based program for the secondary prevention of ischemic stroke in China. Stroke; a journal of cerebral circulation. 2014;45(2):515–9. Epub 2014/01/05. 10.1161/STROKEAHA.113.001424 . 24385269

[35] Yusuf S, Pais P, Sigamani A, Xavier D, Afzal R, Gao P, et al Comparison of risk factor reduction and tolerability of a full-dose polypill (with potassium) versus low-dose polypill (polycap) in individuals at high risk of cardiovascular diseases: the Second Indian Polycap Study (TIPS-2) investigators. Circulation Cardiovascular quality and outcomes. 2012;5(4):463–71. Epub 2012/07/13. 10.1161/circoutcomes.111.963637 . 22787067

[36] Investigators G. Randomised trial of telephone intervention in chronic heart failure: DIAL trial. Bmj. 2005;331(7514):425 Epub 2005/08/03. 10.1136/bmj.38516.398067.E0 16061499 PMC1188105

[37] Ferrante D, Varini S, Macchia A, Soifer S, Badra R, Nul D, et al Long-term results after a telephone intervention in chronic heart failure: DIAL (Randomized Trial of Phone Intervention in Chronic Heart Failure) follow-up. Journal of the American College of Cardiology. 2010;56(5):372–8. Epub 2010/07/24. 10.1016/j.jacc.2010.03.049 . 20650358

[38] Khonsari S, Subramanian P, Chinna K, Latif LA, Ling LW, Gholami O. Effect of a reminder system using an automated short message service on medication adherence following acute coronary syndrome. European journal of cardiovascular nursing: journal of the Working Group on Cardiovascular Nursing of the European Society of Cardiology. 2014 Epub 2014/02/05. 10.1177/1474515114521910 . 24491349

[39] Ortega KC, Gusmão JLd, Pierin AMG, Nishiura JL, Ignez EC, Segre CA, et al How to avoid discontinuation of antihypertensive treatment: The experience in São Paulo, Brazil. Clinics. 2010;65(9):857–63. 10.1590/s1807-59322010000900008 21049213 PMC2974815

[40] Piette JD, Datwani H, Gaudioso S, Foster SM, Westphal J, Perry W, et al Hypertension Management Using Mobile Technology and Home Blood Pressure Monitoring: Results of a Randomized Trial in Two Low/Middle-Income Countries. Telemedicine and e-Health. 2012;18(8):613–20. 10.1089/tmj.2011.0271 23061642 PMC4361160

[41] Labhardt ND, Balo J-R, Ndam M, Manga E, Stoll B. Improved retention rates with low-cost interventions in hypertension and diabetes management in a rural African environment of nurse-led care: a cluster-randomised trial. Tropical Medicine & International Health. 2011;16(10):1276–84. 10.1111/j.1365-3156.2011.02827.x 21733046

[42] Obreli-Neto PR, Guidoni CM, de Oliveira Baldoni A, Pilger D, Cruciol-Souza JM, Gaeti-Franco WP, et al Effect of a 36-month pharmaceutical care program on pharmacotherapy adherence in elderly diabetic and hypertensive patients. International journal of clinical pharmacy. 2011;33(4):642–9. Epub 2011/05/06. 10.1007/s11096-011-9518-x . 21544559

[43] Qureshi NN, Hatcher J, Chaturvedi N, Jafar TH, Hypertension Research G. Effect of general practitioner education on adherence to antihypertensive drugs: cluster randomised controlled trial. Bmj. 2007;335(7628):1030 Epub 2007/11/10. 10.1136/bmj.39360.617986.AE 17991935 PMC2078673

[44] Anchala R, Kaptoge S, Pant H, Di Angelantonio E, Franco OH, Prabhakaran D. Evaluation of effectiveness and cost-effectiveness of a clinical decision support system in managing hypertension in resource constrained primary health care settings: results from a cluster randomized trial. Journal of the American Heart Association. 2015;4(1):e001213 Epub 2015/01/07. 10.1161/jaha.114.001213 ; PubMed Central PMCID: PMCPmc4330052. 25559011 PMC4330052

[45] Mendis S, Johnston SC, Fan W, Oladapo O, Cameron A, Faramawi MF. Cardiovascular risk management and its impact on hypertension control in primary care in low-resource settings: a cluster-randomized trial. Bulletin of the World Health Organization. 2010;88(6):412–9. Epub 2010/06/12. 10.2471/BLT.08.062364 20539854 PMC2878142

[46] Reutens AT, Hutchinson R, Van Binh T, Cockram C, Deerochanawong C, Ho L-T, et al The GIANT study, a cluster-randomised controlled trial of efficacy of education of doctors about type 2 diabetes mellitus management guidelines in primary care practice. Diabetes Research and Clinical Practice. 2012;98(1):38–45. 10.1016/j.diabres.2012.06.002 22784926

[47] Steyn K, Lombard C, Gwebushe N, Fourie JM, Everett-Murphy K, Zwarenstein M, et al Implementation of national guidelines, incorporated within structured diabetes and hypertension records at primary level care in Cape Town, South Africa: a randomised controlled trial. Global health action. 2013;6:20796 Epub 2013/09/28. 10.3402/gha.v6i0.20796 24070181 PMC3784670

[48] Alhalaiqa F, Deane KH, Nawafleh AH, Clark A, Gray R. Adherence therapy for medication non-compliant patients with hypertension: a randomised controlled trial. Journal of human hypertension. 2012;26(2):117–26. Epub 2011/02/18. 10.1038/jhh.2010.133 21326328 PMC3257548

[49] Mohammadi E, Abedi HA, Jalali F, Gofranipour F, Kazemnejad A. Evaluation of 'partnership care model' in the control of hypertension. International journal of nursing practice. 2006;12(3):153–9. Epub 2006/05/06. 10.1111/j.1440-172X.2006.00563.x . 16674782

[50] Ahmadipour H, Farajzadegan Z, Kachoei A, Pirdehghan A. Secondary prevention by enhancing adherence in diabetic patients. Int J Prev Med. 2010;1(1):50–5. Epub 2010/01/01. ; PubMed Central PMCID: PMCPmc3075488. 21677766 PMC3075488

[51] Tan MY, Magarey JM, Chee SS, Lee LF, Tan MH. A brief structured education programme enhances self-care practices and improves glycaemic control in Malaysians with poorly controlled diabetes. Health education research. 2011;26(5):896–907. Epub 2011/07/01. 10.1093/her/cyr047 . 21715653

[52] Arevian M. The significance of a collaborative practice model in delivering care to chronically ill patients: a case study of managing diabetes mellitus in a primary health care center. Journal of interprofessional care. 2005;19(5):444–51. Epub 2005/11/26. 10.1080/13561820500215095 . 16308168

[53] Andryukhin A, Frolova E, Vaes B, Degryse J. The impact of a nurse-led care programme on events and physical and psychosocial parameters in patients with heart failure with preserved ejection fraction: a randomized clinical trial in primary care in Russia. The European journal of general practice. 2010;16(4):205–14. Epub 2010/11/16. 10.3109/13814788.2010.527938 . 21073267

[54] Bocchi EA, Cruz F, Guimaraes G, Pinho Moreira LF, Issa VS, Ayub Ferreira SM, et al Long-term prospective, randomized, controlled study using repetitive education at six-month intervals and monitoring for adherence in heart failure outpatients: the REMADHE trial. Circulation Heart failure. 2008;1(2):115–24. Epub 2008/07/01. 10.1161/CIRCHEARTFAILURE.107.744870 . 19808281

[55] Organization WH. Task shifting: global recommendations and guidelines. 2008.

[56] Macharia WM, Njeru EK, Muli-Musiime F, Nantulya V. Severe road traffic injuries in Kenya, quality of care and access. African health sciences. 2009;9(2):118–24. Epub 2009/08/05. ; PubMed Central PMCID: PMCPmc2707053. 19652745 PMC2707053

[57] Sekhri N, Feachem R, Ni A. Public-private integrated partnerships demonstrate the potential to improve health care access, quality, and efficiency. Health affairs. 2011;30(8):1498–507. Epub 2011/08/09. 10.1377/hlthaff.2010.0461 . 21821566

[58] Yusuf S, Islam S, Chow CK, Rangarajan S, Dagenais G, Diaz R, et al Use of secondary prevention drugs for cardiovascular disease in the community in high-income, middle-income, and low-income countries (the PURE Study): a prospective epidemiological survey. The Lancet. 2011;378(9798):1231–43. 10.1016/s0140-6736(11)61215-4 21872920

[59] Rabkin M, Kruk ME, El-Sadr WM. HIV, aging and continuity care: strengthening health systems to support services for noncommunicable diseases in low-income countries. AIDS (London, England). 2012;26 Suppl 1:S77–83. Epub 2012/09/22. 10.1097/QAD.0b013e3283558430 . 22781180

[60] Binanay CA, Akwanalo CO, Aruasa W, Barasa FA, Corey GR, Crowe S, et al Building Sustainable Capacity for Cardiovascular Care at a Public Hospital in Western Kenya. Journal of the American College of Cardiology. 2015;66(22):2550–60. 10.1016/j.jacc.2015.09.086 26653630 PMC4680855

[61] Vedanthan R, Kamano JH, Bloomfield GS, Manji I, Pastakia S, Kimaiyo SN. Engaging the Entire Care Cascade in Western Kenya. Global heart. 2015;10(4):313–7. 10.1016/j.gheart.2015.09.003 26704963 PMC4691279

[62] Hemming K, Haines TP, Chilton PJ, Girling AJ, Lilford RJ. The stepped wedge cluster randomised trial: rationale, design, analysis, and reporting. Bmj. 2015;350:h391 Epub 2015/02/11. 10.1136/bmj.h391 . 25662947

[63] Ranasinghe I, Rong Y, Du X, Wang Y, Gao R, Patel A, et al System barriers to the evidence-based care of acute coronary syndrome patients in China: qualitative analysis. Circulation Cardiovascular quality and outcomes. 2014;7(2):209–16. Epub 2014/03/13. 10.1161/CIRCOUTCOMES.113.000527 . 24619324

[64] Gaziano TA, Bertram M, Tollman SM, Hofman KJ. Hypertension education and adherence in South Africa: a cost-effectiveness analysis of community health workers. BMC public health. 2014;14:240 Epub 2014/03/13. 10.1186/1471-2458-14-240 ; PubMed Central PMCID: PMCPmc3973979. 24606986 PMC3973979

[65] FDA. Briefing Document: Cardiovascular and Renal Drugs Advisory Committee Meeting. 2014.

[66] International Telecommunications Union. Geneva S. The World in 2014: ICT Facts and Figures. 2014. Epub April 2014.

[67] de Jongh T, Gurol-Urganci I, Vodopivec-Jamsek V, Car J, Atun R. Mobile phone messaging for facilitating self-management of long-term illnesses. The Cochrane database of systematic reviews. 2012;12:Cd007459 Epub 2012/12/14. 10.1002/14651858.CD007459.pub2 . 23235644 PMC6486189

[68] Proctor E, Silmere H, Raghavan R, Hovmand P, Aarons G, Bunger A, et al Outcomes for Implementation Research: Conceptual Distinctions, Measurement Challenges, and Research Agenda. Administration and Policy in Mental Health. 2011;38(2):65–76. 10.1007/s10488-010-0319-7 . 20957426 PMC3068522

