# Supplementary figures and images for: Quality Improvement for Cardiovascular Disease Care in Low- and Middle-Income Countries: A Systematic Review
Source: PLoS One. 2016 Jun 14;11(6):e0157036. doi: 10.1371/journal.pone.0157036 (PMC4907518; doi:10.1371/journal.pone.0157036)

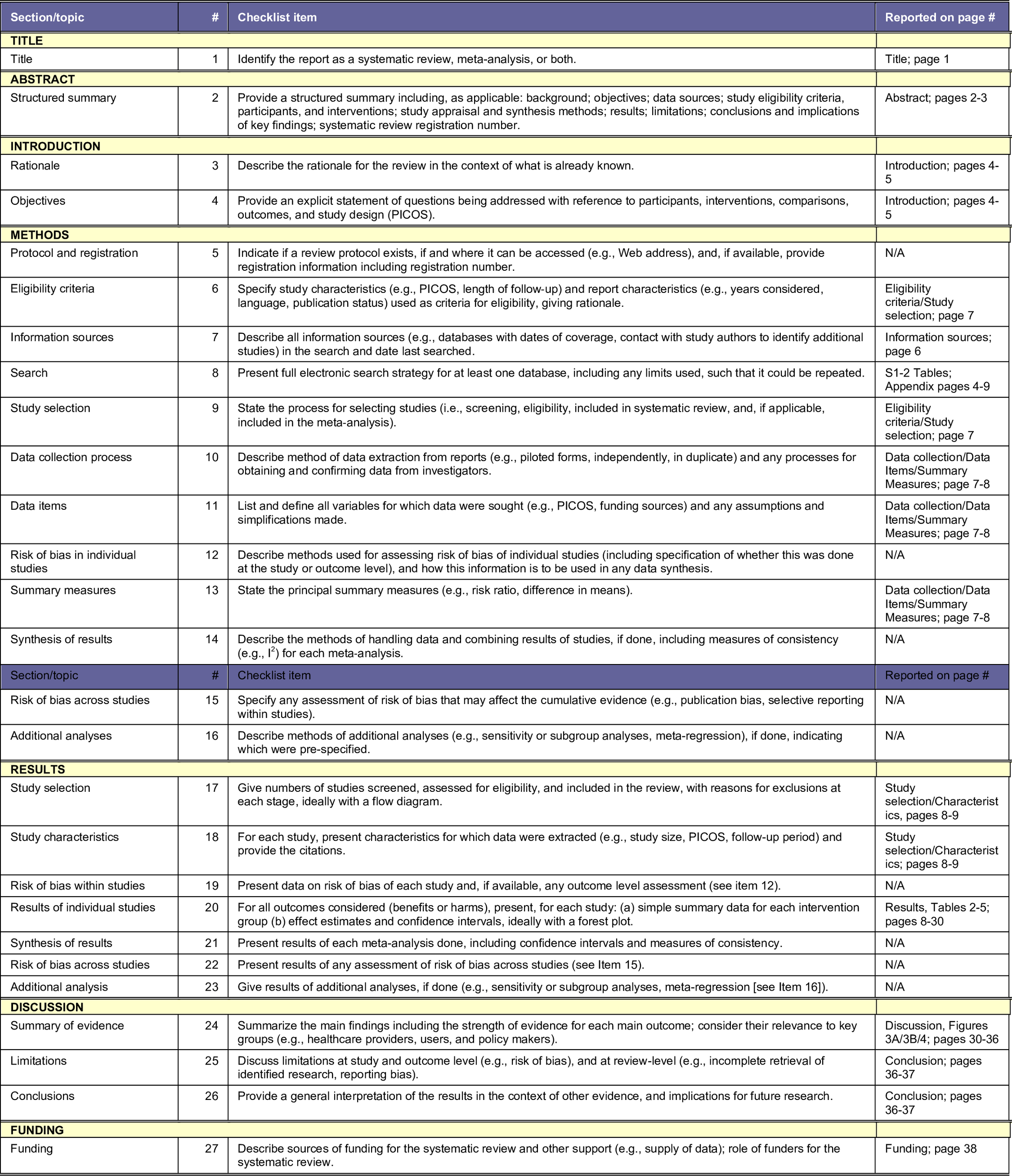

Supplement: S1 Fig — (TIF) [file pone.0157036.s001.tif]

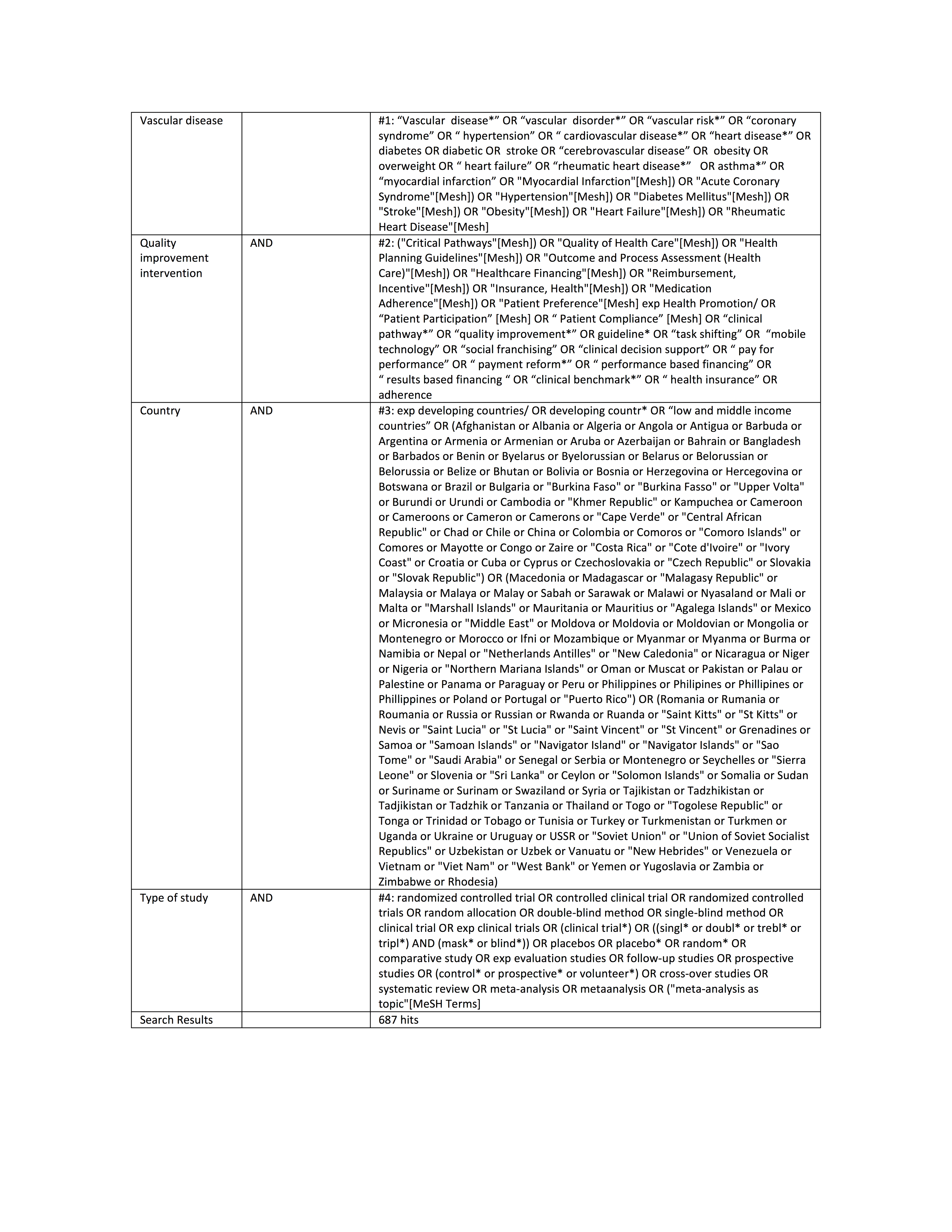

Supplement: S1 Table — (TIF) [file pone.0157036.s002.tif]

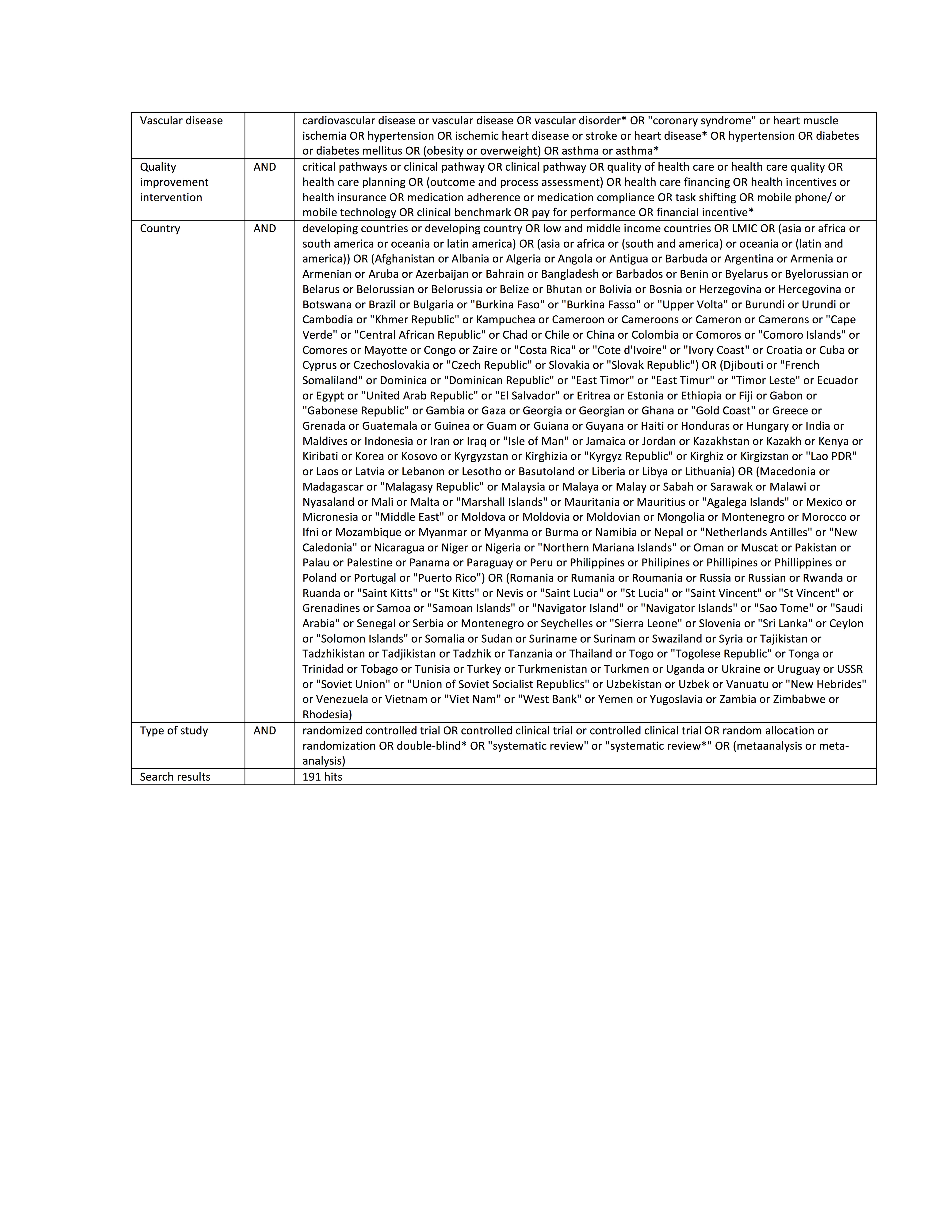

Supplement: S2 Table — (TIF) [file pone.0157036.s003.tif]
